# Nigerian medicinal plants with potential anticancer activity—a review

**DOI:** 10.37349/etat.2024.00282

**Published:** 2024-12-09

**Authors:** Mansurah A. Abdulazeez, Hiba A. Jasim, Temidayo D. Popoola, Saheed O. Benson, Jiradej Manosroi, Abdullahi B. Sallau, Musa A. Tabari, Amos A. Fatokun

**Affiliations:** NGO Praeventio, Estonia; ^1^Center for Biotechnology Research, Bayero University, Kano, Kano State, Nigeria; ^2^Center for Natural Products Discovery (CNPD), School of Pharmacy and Biomolecular Sciences, Liverpool John Moores University, Liverpool L3 3AF, UK; ^3^Department of Biology, College of Education for Pure Sciences, University of Anbar, Ramadi, Iraq; ^4^Department of Cosmetic Technology, Faculty of Engineering, North-Chiang Mai University, Chiang Mai, Thailand; ^5^Department of Biochemistry, Faculty of Life Sciences, Ahmadu Bello University, Zaria, Nigeria; ^6^Department of Radiology, Barau Dikko Teaching Hospital (BDTH), Kaduna State University (KASU), Kaduna, Nigeria

**Keywords:** Cancer, chemotherapy, cytotoxicity, ethnomedicine, medicinal plants, Nigeria

## Abstract

Despite the fact that life expectancies are increasing and the burden of infectious diseases is decreasing, global cancer incidence rates are on the rise. Cancer outcome metrics are dismal for low- and middle-income countries (LMICs), including sub-Saharan Africa, where adequate resources and infrastructure for cancer care and control are lacking. Nigeria, the most populous country in Africa, exemplifies the miserable situation. However, the investigation of medicinal plants for better and safer anti-cancer drugs has now increased tremendously. While scientific evidence is emerging of the potential of some constituents of medicinal plants used in traditional medicine in Nigeria to have anti-cancer effects, there is now a critical need for platforms that integrate ethnomedicinal information on such plants with emerging scientific data on them, to support and accelerate the discovery and development of more efficacious and safer anti-cancer drugs and recipes. Thus, this review highlights the scientific evidence to date for the anti-cancer potential of plants commonly used in traditional medicine to treat cancers in Nigeria. Scientific databases such as PubMed, Science Direct, Scopus, Google Scholar, and Web of Science, as well as related sources, were searched to retrieve relevant information on anti-cancer medicinal plants. Ethnobotanical/ethnomedicinal details of the identified plants were then linked with the available scientific data on their anti-cancer potential, including the cytotoxicity to cancer and normal cells of the extracts and constituent compounds responsible for the activity. This annotated chronicle of Nigerian medicinal plants with potential anticancer activity is a great resource for all stakeholders in the prevention and management of cancers.

## Introduction

Cancer could be simply described as the uncontrolled growth and spread of abnormal cells, which starts in one organ or tissue called the primary site, and, if undetected or cannot be controlled through treatment, spreads (metastasizes) to other organs in the body. It is a heterogeneous disease with a significant genetic component, featuring changes to at least three categories of genes involved in tumorigenesis: oncogenes, tumor suppressor genes, and stability genes [1]. There are more than 100 types of cancer, named based on their primary site, e.g., breast cancer starts in the breast, lung cancer starts in the lung, and ovarian cancer starts in the ovaries [2]. Generally, cancers can be carcinomas, when they arise from the epithelial cell lining; sarcoma, when they arise from mesodermal cells lining the muscles, bones, cartilage, and connective tissue; lymphoma, when they arise from cells of the immune system; and leukemia, when they arise from cells of the bone marrow [3, 4].

Cancer is second only to cardiovascular disease as the main cause of death in developed countries and is an increasingly important public health problem in developing countries, including African countries. From an estimated 12 million new cases and 7.6 million deaths in 2008 to 19.3 million new cases and 10 million deaths in 2020 [5] the incidence of cancer worldwide is expected to rise to 26.4 million with 17 million deaths by 2030, with 70% of the deaths expected to occur in the developing world [6–8]. Globally, breast cancer is the most commonly diagnosed cancer, and the most common among women with over 2.2 million cases in 2020, while lung cancer is the most common cancer in men (over 1.4 million cases), and the leading cause of death, with an approximate 1.8 million deaths in 2020 [5]. The incidence rate for all cancers worldwide was 19% higher in men than women, although rates varied across regions, reflecting differences in risk factors, cancer prevention, and early detection methods [5].

Nigeria, the largest and most populous developing country in Africa, exemplifies the current dismal state of cancer in Africa, with more than 120,000 new cases of cancer and up to 71,000 deaths in 2020. The disease is wreaking great havoc and its damaging effect cannot be overestimated [8]. This unfortunate situation has been attributed to the poor oncology services and poor radiation therapy facilities that are accessible to only 15% of those in need of radiotherapy. In addition, there are problems of inappropriate or ill-advised choice of readily available and accessible herbal and spiritual treatment options, lack of awareness and ill-informed perceptions about cancer, prolonged denial, suboptimal numbers of trained pathologists and physicians in women’s health, failure in stewardship by government in ensuring equitable distribution of health facilities and services, low wages for healthcare workers, etc., all of which have significantly contributed to the low survival rates [9].

According to the World Health Organization’s (WHO) global snapshot for cancer control in 2015, the availability of cancer treatment services: cancer centers, surgeries, and subsidized chemotherapy, was lowest in Africa (just above 35%) [10]. A majority of those who cannot afford these drugs depend on traditional medicines derived from natural products, which could be considered the mainstay of healthcare in many developing countries [11]. Natural products are a rich reservoir of bioactive constituents isolated from microbes, plants, and other living organisms with therapeutic potentials. They are recognized as genuine sources of drugs used to treat several human ailments, including cancers. Examples include vincristine, irinotecan, etoposide, and paclitaxel from plants, actinomycin D and mitomycin C from bacteria, and marine-derived bleomycin [12]. Several of the currently available anticancer drugs were derived from natural/plant sources. These medicinal plants are vital in the search for novel anticancer compounds due to the pharmacological actions of their diverse chemical constituents in the human body [13]. Although many anticancer drugs have been derived from plants, many more anticancer medicinal plants are yet to be discovered [13].

In Nigeria, medicinal plant extracts are widely used as important sources of chemotherapeutic agents despite the use of synthetic drugs by a majority of the population. Many Nigerians, especially those living in rural areas depend on these plants for the treatment of various diseases, including cancer. Although the therapeutic potency of some medicinal plants against several types of cancers has been established [14], the search for new anticancer agents is still necessary to develop drugs that are less toxic and more effective and to increase their variety and availability [15].

## Medicinal plants as an alternative treatment regimen

Plants have been used as herbal medicines since the beginning of human history as dietary supplements as well as in the therapy and management of several diseases, including cancer. In fact, before the 20th century, about 80% of all medicines for the treatment of various diseases were products of medicinal plants [16]. Fruits, vegetables, and spices, being primary sources of naturally occurring nutrients essential for human health, are now popular among consumers due to their health benefits. Also, the number of medicinal plants being used in healthcare has increased worldwide, as they have been shown to contain several bioactive compounds possessing various medicinal properties, such as antioxidant, anti-inflammatory, antibacterial, antimutagenic, antidiabetic, and anticarcinogenic activities. These make them attractive potential agents for preventing or treating diseases in humans [17]. It is noteworthy that a relatively small percentage of these plants have been or are being evaluated for their potential as therapeutic agents, even though the potential of most plants as safe sources of food has been exploited. Thus, there are research gaps to fill by examining food crops, especially vegetables, for their potential development as therapeutic agents [18].

Herbal medicine, considered “alternative medicine” in contrast to current conventional (western) medicine, is based on the use of plants or plant extracts to treat diseases and promote health and has gained more prominence over the last century. Alternative medicine, also called complementary and alternative medicine, holistic medicine, complementary medicine, natural medicine, traditional medicine, natural therapies, and unorthodox medicine [19] is defined as a group of diverse medical and healthcare systems, practices, and products that are not presently considered to be part of conventional medicine [20]. It is also defined as therapies not taught in medical schools, not used in hospitals, and not reimbursed by medical insurance companies [21].

Over the past decades, the relevance of herbal medicine has been appreciated due to its contribution to both health and international commerce. Herbal medicine is now widely accepted, as reflected in its increased patronage and publicity [22]. Biodiversity and traditional medical knowledge of these herbal medicines have led to the development of almost 70% of the drugs currently in use [16]. Also, approximately 700 natural products or natural product-derived New Chemical Entities (NCEs) were approved between 1981 and 2010 [16]. These natural products have proved a valuable source of drug leads for many years as a result of the degree of their chemical diversity. The testing of their extracts against biological targets has been widely undertaken in the pharmaceutical industry [23].

In Africa and other developing countries, the high costs of modern drugs contribute to the continuous use of plants [24]. About 80% of the world’s population living in rural areas depend on medicinal plants [25] prescribed by herbal practitioners to treat several diseases. In Nigeria, it is well known that for several decades, local communities have used herbal medicines for the treatment of several diseases. Also, traditional medicine is regarded as part of the cultural heritage and is acceptable to the majority of the populace [18]. According to the WHO, plant-based treatments are still in use as the main source of medicine in some developed and most developing countries [26]. Parts of India and China cultivate medicinal plants on a large scale to keep up with increasing demands for alternative drugs, for example, due to the discovery of potent cytotoxic agents attributed to Asian and Ayurvedic Indian traditional medicines [27].

## Plants for cancer treatment

Reports by the WHO [28] and the World Cancer Research Fund [29] have attributed the increasing incidence of cancer to diet, environment, and carcinogenic virus infections. Despite current improvements in cancer prevention, diagnosis, and treatment strategies, such as chemotherapy, radiotherapy, and surgery, mortality from the disease remains high. Some of the treatments have been useful in some and not in all cancer types [30]. The side effects from some drugs compromise continued treatment, making affected patients resort to herbal medicines. As a result, renewed and concerted efforts are geared towards the discovery and development of safer and well-tolerated anticancer drugs from natural products, mainly plants. New targets for anticancer drug development are rapidly emerging in the post-genomic era, and improvements in high-throughput, small-molecule screens, protein structure determination, and combinatorial chemistry have hugely contributed to the generation of new drug targets [30].

Currently, scientific evidence is still growing to support claims that herbal medicine can treat or prevent cancer. However, as research and development of more effective and less toxic drugs by the pharmaceutical industry remain valuable, there is a focus on exploring medicinal plants to discover novel, potential anticancer compounds with little or no unwanted effects [31]. Several lead structures isolated from plants due to their diverse biosynthetic pathways have been used in drug development, and recent investigations of natural compounds in the search for anticancer drugs have produced promising results. Almost 60% of drugs currently used for cancer treatment were isolated from natural products, with plants contributing considerably: vinca alkaloids, taxanes, camptothecin, combretastin, podophyllotoxin, geniposide and their derivatives, colchine, etc. The most common plant-derived anticancer compounds of medical importance include vincristine, vinblastine, and taxanes, such as docetaxel and taxol [12].

**Figure 1:**
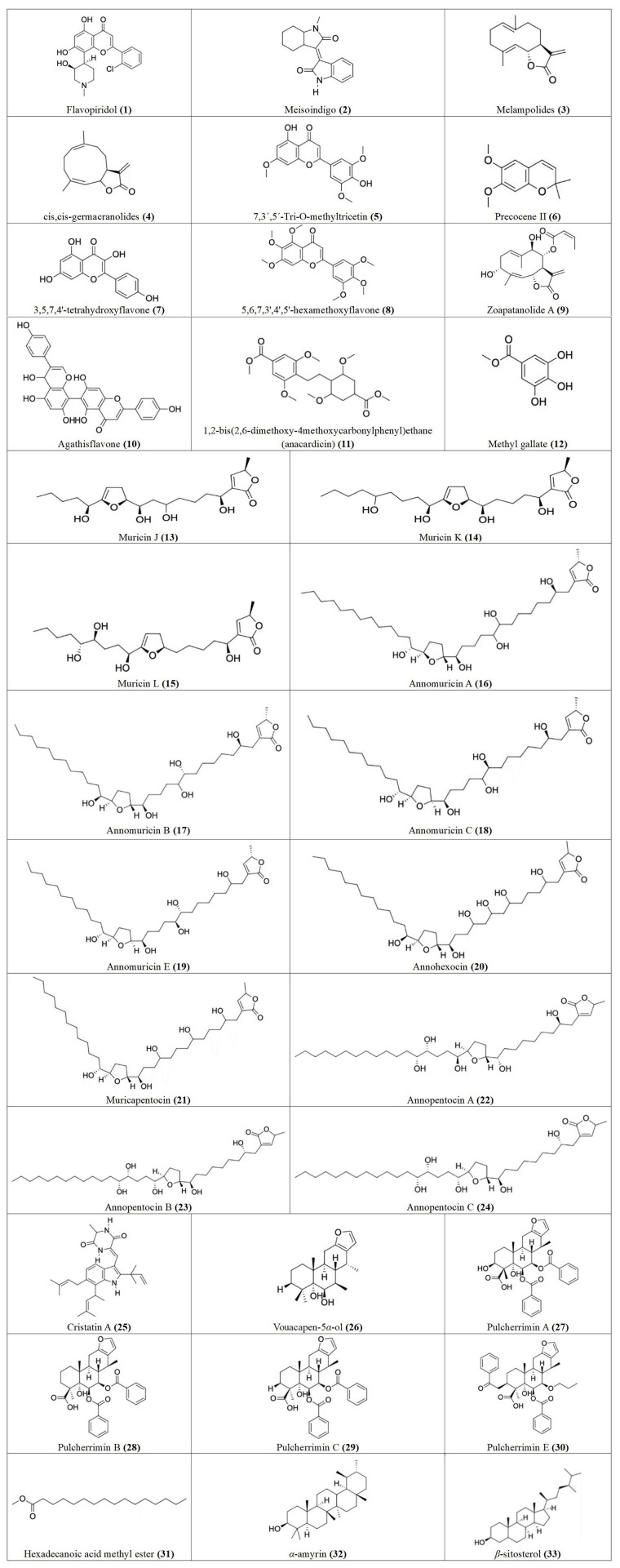
Chemical structures of Table 1 compounds **1–33**

Vinca alkaloids, also known as catharanthus alkaloids, include vinblastine, vincristine, and its derivatives. While vinblastine was the first alkaloid isolated from the periwinkle plant, vincristine, and its derivatives were formed from the biosynthesis of catharanthine and vindoline and are present in pink *Catharanthus roseus* [12]. Vinca alkaloids are used either alone or together with other drugs to treat breast cancer, osteosarcoma, and acute lymphocytic leukemia. The activity of vinca alkaloids is comparable to that of taxanes, e.g., taxol, isolated from the bark of *Taxus baccata* and *Taxus brevifolia* tree needles. Taxol is one of the most effective drugs for the treatment of breast, ovarian, and squamous cell carcinoma of the head and neck [12]. Some of these compounds remain the cornerstone of cancer therapy and will continue to play crucial roles in the foreseeable future. There are now a series of derivatives of these compounds with improved properties, e.g., Nab-paclitaxel, a taxol derivative, approved for the treatment of metastatic breast cancer [13]; vinorelbine, a semisynthetic vinca alkaloid used to treat non-small cell lung and breast cancers; and vindesine which is currently in phase II clinical trials for the treatment of non-small cell lung cancer, hepatocellular cancers, and leukemia [12]. Many more compounds with promising anticancer properties have been isolated from plants, for example, flavopiridol **(1)**, isolated from the Indian tree *Dysoxylum binectariferum*, and meisoindigo **(2)**, isolated from the Chinese plant *Indigofera tinctoria* (see Figure 1), have been shown to exhibit anticancer effects, with less undesirable toxicity than conventional drugs [15, 32, 33].

Although cancer incidence is expected to increase in the future, causing a strain on health care, appropriate allocation of resources for research into early diagnosis, curative and palliative care, and drug development would go a long way to bring about a positive change [34].

## Nigeria’s biodiversity

Nigeria, with a landmass of 923,768 sq. km, is situated entirely within the tropical zone and located between latitudes 4° and 14° north of the equator and longitudes 3° and 15° east. The country’s topography ranges from lowlands along the coast and in the lower Niger Valley to high plateaus in the north and mountains along the eastern border. Much of the country is laced with productive rivers. Nigeria’s ecology varies from tropical forest in the south to dry savanna in the far north, yielding a diverse mix of plant and animal life [35].

The rich African biodiversity exemplifies the species, distributions, and number of medicinal plants available. Africa is home to over 57,000 species of the world’s flora, but only about 5,000 are used traditionally or combined as recipes for their medicinal properties, with most unexplored [35]. This makes drug discovery of African plants of relevant interest. Nigeria, the most populous African country, possesses diverse plant species with a history of traditional use spanning many centuries, many of which are yet to receive enough research attention to make local sourcing of pharmaceutical products a reality. There is no doubt the country has the potential to be a veritable source of pharmaceuticals and other therapeutic materials [36]. Awareness by international bodies such as the International Genetic Resources Institute (IGRI), Consultative Group on International Agriculture Research, the Global Forum on Agriculture Research, and the International Centre for Underutilized Crops has led to an increase in research aimed at sourcing for pharmaceuticals that would be beneficial to the country and the world at large [18]. Also, to promote traditional medicine, the Nigerian government launched a new, high-profile committee on traditional medicine to help develop, promote, and commercialize traditional medicine products, an endeavor that could generate for the country at least US$1 billion in the first ten years [37]. Thus, it is pertinent to provide information that would reveal the potential of these plants and justify their use for a successful fight against cancer.

## Methodology

### Search strategy

Scientific databases such as PubMed, Science Direct, Scopus, Google Scholar, and Web of Knowledge were searched to retrieve publications using the key terms: cancer, cytotoxicity, plants, and Nigeria. Articles related to the subject and published from 1999 to 2022 were used and included in the reference list.

### Inclusion and exclusion criteria

Both review and original data articles on medicinal plants for cancer treatment in Nigeria were considered. Plant species were taxonomically validated; the Latin scientific name and family were confirmed using The Plant List site (http://www.theplantlist.org). Ethnopharmacological articles with methodological bias in terms of sample superiority and validity of the species were excluded.

## Nigerian medicinal plants with potential anticancer (cytotoxic) effects

**Figure 2:**
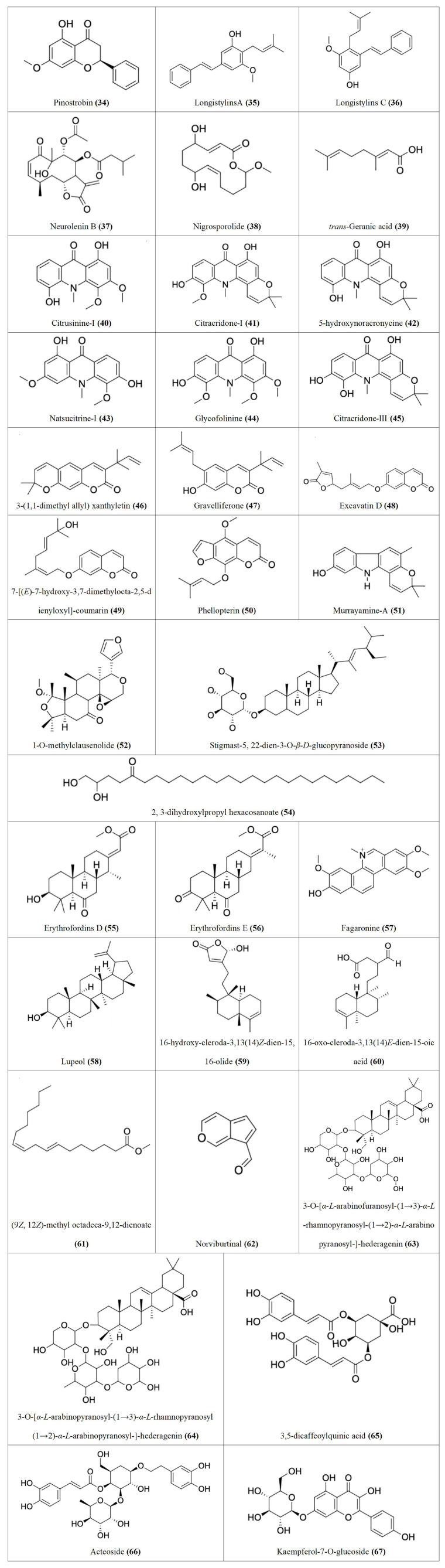
Chemical structures of Table 1 compounds **34–67**

**Figure 3:**
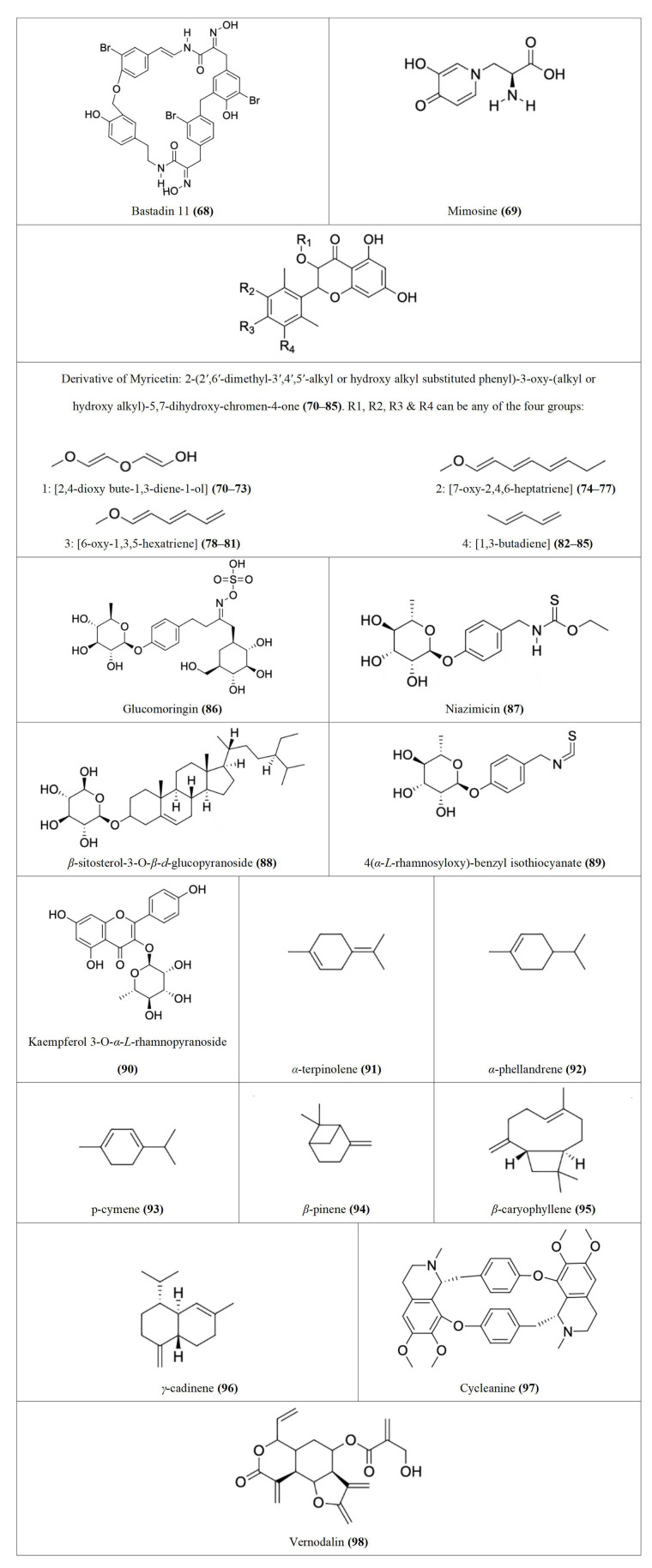
Chemical structures of Table 1 compounds **68–98**

Following our search, several Nigerian plants eliciting cytotoxic activity and their bioactive constituents were identified, as shown in Table 1. The chemical structures of the bioactive constituents are shown in Figure 1, Figure 2, and Figure 3.

**Table 1:** Cytotoxic effects and bioactive constituents of some Nigerian medicinal plants [see Figures 1, Figure 2, and Figure 3 for the chemical structures (of the compounds) indicated in the table by numbers in the brackets]

**Botanical name/family/plantpart(s)**	**Traditional use(s)**	**Potential bioactive compound(s)**	**Cytotoxic effects**
*Acanthospermum hispidum* DC Asteraceae Flowering shoots, aerial parts	Cancer	Melampolides **(3)** Cis,cis-germacranolides **(4)**	Methanol extract on COR-L23 (IC_50_: 8.87 ± 0.90 μg/mL), MCF-7 (IC_50_: 13.50 ± 1.00 μg/mL) and C32 (IC_50_: 13.54 ± 0.8 μg/mL) cell lines [38] Methanol fraction on RD cell line (IC_50_: 19.65 ± 1.23 μg/mL) [39] Methanol extract on 5637 (IC_50_: 9.37 ± 1.98 μg/mL), MCF-7 (IC_50_: 19.92 ± 8.94 μg/mL) and A-427 (IC_50_: 16.70 ± 2.32 μg/mL) cell lines [40]
*Afzelia africana* Sm. Ex Pers Fabaceae Leaves, stem bark	Pain, malaria, gonorrhea, and leprosy		Methanol extract on HEK (IC_50_: 0.65 μg/mL) [41] Methanol extract on HEK (IC_50_: 0.54 μg/mL) [41] Ethyl acetate extract on PC-3 (IC_50_: 12.5 μg/mL) and MCF-7 (IC_50_: 14.5 μg/mL) [42]
*Ageratum conyzoides* Linn. Asteraceae	Epilepsy, wounds, insect repellent, burns, cuts and sore and throat infections	7,3',5'-Tri-O-methyltricetin **(5)** Precocene II **(6)** 3,5,7,4'-tetrahydroxyflavone **(7)** 5,6,7,3',4',5'-hexamethoxyflavone **(8)**	Petroleum ether extract on SGC-7901 (IC_50_: 13.77 μg/mL), A549 (IC_50_: 14.06 μg/mL), and P-388 (IC_50_: 0.71 μg/mL) cancer cell lines [43] Ethyl acetate extract on A-549 (IC_50_: 0.68 μg/mL), SGC-7901 (IC_50_: 14.38 μg/mL), P-388 (IC_50_: 0.0003 μg/mL), and DU-145 (IC_50_: 9.90 μg/mL) cancer cell lines [43] Ethanol extract on P-388 (IC_50_: 1.73 μg/mL) [43] 7,3',5'-Tri-O-methyltricetin **(5)** (IC_50_: 12.8 µM), Precocene II **(6)** (IC_50_: 24.8 µM), 3,5,7,4'-tetrahydroxyflavone **(7)** (IC_50_: 3.5 µM) and 5,6,7,3',4',5'-hexamethoxyflavone **(8)** (IC_50_: 7.8 μM) on P-388 cell line [44] Precocene II **(6)** (IC_50_: 61 μM) on the HT-29 cancer cell line [44]
*Allanblackia floribunda* Oliv Guttiferae	Malaria, dysentery		Methanol extract on BT-549 (IC_50_: 14.7 ± 0.23 μg/mL), BT-20 (IC_50_: 48.3 ± 2.90 μg/mL), PC-3 (IC_50_: 29.4 ± 0.69 μg/mL) and SW-480 (IC_50_: 57.1 ± 1.16 μg/mL) [45]
*Anacardium occidentale* L. Anacardiaceae Leaves	Cancer	Zoapatanolide A **(9)** Agathisflavone **(10)** 1,2-bis(2,6-dimethoxy-4-methoxycarbonylphenyl)ethane (anacardicin) **(11)** Methyl gallate **(12)**	Acetone extract on HeLa cells (IC_50_: 36.2 ± 9.8 μM) [46]
*Annona muricata* L. Annonaceae Leaves, roots, twigs	Cancer, fever, diarrhea, diabetes, headaches, gastric disorders, rheumatism, arthritic pain	Muricin J **(13)** Muricin K **(14)** Muricin L **(15)** Annomuricin A **(16)** Annomuricin B **(17)** Annomuricin C **(18)** Annomuricin E **(19)** Annohexocin **(20)** Muricapentocin **(21)** Annopentocin A **(22)** Annopentocin B **(23)** Annopentocin C **(24)**	Acetogenin-enriched fraction on PC-3 (IC_50_: 57 μg/mL) [47] Ethanol extract on PC-3 (IC_50_: 63 μg/mL) [47], HL-60 (IC_50_: 14 ± 2.4 μg/mL), EACC (IC_50_: 335.85 µg/mL), MDA-MB-231 (IC_50_: 248.77 µg/mL) and SKBR3 (IC_50_: 202.33 µg/mL) cancer cell lines [48] Aqueous extract on MCF-7 (IC_50_: 221.67 ± 1.67 μg/mL), MDA-MB-231 (IC_50_: 350 ± 5.77 μg/mL), and 4T1 (IC_50_: 251.67 ± 6.01 μg/mL) cancer cells [49] Ethyl acetate extract on MCF-7 (IC_50_: 6.39 ± 0.43 μg/mL), MDA-MB-231 (IC_50_: 11.36 ± 0.67 μg/mL), A549 (IC_50_: 5.09 ± 0.41 μg/mL), HepG2 (IC_50_: 9.3 ± 0.91 μg/mL), WRL-68 (IC_50_: 47.10 ± 1.23 μg/mL) [50], HCT-116 (IC_50_: 8.98 ± 1.24 μg/mL) and HT-29 (IC_50_: 11.43 ± 1.87 μg/mL) cancer cells [51] Ethanol extract on HL-60 (IC_50_: 9 ± 0.8 μg/mL) [52] Ethanol extract on HL-60 (IC_50_: 49 ± 3.2 μg/mL) [52]
*Bidens pilosa* L Compositae	Ear infection, cough, diarrhea		Methanol extract on BT-549 (IC_50_: 43.1 ± 6.09 μg/mL), BT-20 (IC_50_: 53.7 ± 2.16 μg/mL), PC-3 (IC_50_: 47.7 ± 2.69 μg/mL) and Jurkat (IC_50_: 75.6 ± 1.06 μg/mL) [45]
*Breynia nivo*s (W.Bull) Small Phyllanthaceae Leaves	Headaches, toothaches, tooth infections, fever and malaria	Cristatin A **(25)**	Cristatin A **(25)** on L5178Y mouse lymphoma cell line (IC_50_: 13.9 μM) [53] Methanol extract on HepG2 cell line (38.73% cytotoxic) [54]
*Bryophyllum pinnatum* Lam. Crassulaceae	Respiratory tract infections, antibacterial		Methanol extract on BT-549 (IC_50_: 48.2 ± 1.56 μg/mL), BT-20 (IC_50_: 82.4 ± 0.17 μg/mL) and PC-3 (IC_50_: 48.3 ± 1.05 μg/mL) [45]
*Byrsocarpus coccineus* Schumach. & Thonn. Connaraceae Bark, leaves	Jaundice, pile, gonorrhea, venereal disease, impotence		Methanol extract on BT-549 (IC_50_: 24.6 ± 0.99 μg/mL), BT-20 (IC_50_: 52.9 ± 4.11 μg/mL), PC-3 (IC_50_: 43.7 ± 1.02 μg/mL) and Jurkat (IC_50_: 65.2 ± 0.87 μg/mL) [45] Methanol extract on BT-549 (IC_50_: 18.6 ± 4.85 μg/mL), BT-20 (IC_50_: 31.3 ± 0.53 μg/mL), PC-3 (IC_50_: 29.1 ± 0.64 μg/mL) and Jurkat (IC_50_: 43.4 ± 1.77 μg/mL) [45]
*Caesalpinia pulcherrima* (L.) Sw Fabaceae Roots	Stimulant, emmenagogue, abortifacient, fever, malaria	Vouacapen-5*α*-ol **(26)** Pulcherrimin A **(27)** Pulcherrimin B **(28)** Pulcherrimin C **(29)** Pulcherrimin E **(30)**	Chloroform extract on MCF-7 (IC_50_: 15.65 ± 0.21 μM to 36.49 ± 1.39 μM), HeLa (IC_50_: 7.02 ± 0.31 μM to 27.59 ± 0.26 μM) and PC-3 (IC_50_: 15.64 ± 1.30 μM to 27.59 ± 0.26 μM) cell lines [55].
*Cajanus cajan* (L.) Millsp. Fabaceae Leaves	Smallpox, chicken pox, malaria, breast cancer [45]	Hexadecanoic acid methyl ester **(31)** *α*-Amyrin **(32)** *β*-Sitosterol **(33)** Pinostrobin **(34)** Longistylins A **(35)** Longistylins C **(36)**	Methanol extract on COR-L23 (IC_50_: 9.81 ± 0.00 μg/mL) and MCF-7 (16.08 ± 3.30 μg/mL) cell lines [38] Methanol extract on BT-549 (IC_50_: 56.1 ± 10.09 μg/mL), BT-20 (IC_50_: 56.8 ± 2.60 μg/mL), PC-3 (IC_50_: 50.5 ± 0.76 μg/mL) and SW-480 (IC_50_: 52 ± 0.53 μg/mL) [45]
*Calliandra portoricensis* Jacq. Benth Leguminosae Roots	Cancer, analgesic, anti-ulcerogenic and anticonvulsant	Neurolenin B **(37)** Nigrosporolide **(38)** *trans*-Geranic acid **(39)**	Methanol extract on PC-3 inhibited proliferation by 84% and LNCaP (63%) [56] Methanol fraction on LNCaP (IC_50_: 2.4 ± 0.2 µg/mL), DU-145 (IC_50_: 3.3 ± 0.2 µg/mL) and A549 (IC_50_: 3.6 µg/mL) cancer cells [57] Ethyl acetate fraction on RD cells (IC_50_: 0.82 ± 0.08 μg/mL) [39]
*Citrus aurantium* L. Rutaceae Root bark	Cancer and inflammatory diseases	Citrusinine-I **(40)** Citracridone-I **(41)** 5-hydroxynoracronycine **(42)** Natsucitrine-I **(43)** Glycofolinine **(44)** Citracridone-III **(45)**	Dichloromethane fraction on A549 (IC_50_: 3.88 ± 0.58 μg/mL), HepG2 (IC_50_: 5.73 ± 0.99 μg/mL), MCF-7 (IC_50_: 5.12 ± 0.54 μg/mL) and PC-3 (IC_50_: 4.72 ± 0.23 μg/mL) Methanol fraction on A549 (IC_50_: 88.9 ± 1.23 μg/mL), HepG2 (IC_50_: 92.7 ± 4.11 μg/mL), MCF-7 (IC_50_: 90.6 ± 4.54 μg/mL) and PC-3 (IC_50_: 78.2 ± 2.14 μg/mL) Flavonoid fraction on AGS cells (IC_50_: 99 μg/mL) [58], A549 (IC_50_: 230 μg/mL) [59] and HepG2 (IC_50_: 75 µg/mL) cells [60]
*Clausena anisata* (Willd.) Hook.f. ex Benth Rutaceae Leaves, stem bark	Cancer	3-(1,1-dimethyl allyl) xanthyletin **(46)** Gravelliferone **(47)** Excavatin D **(48)** 7-[(*E*)-7-hydroxy-3,7-dimethylocta-2,5-dienyloxyl]-coumarin **(49)** Phellopterin **(50)**	Methanol fraction on RD cell line (IC_50_: 8.83 ± 0.59 μg/mL) [38] On HeLa cells: IC_50_: 1.14 ± 0.16 μg/ mL **(46)** IC_50_: 1.81 ± 0.09 μg/ mL **(47)** IC_50_: 2.98 ± 0.22 μg/ mL **(48)** IC_50_: 1.27 ± 0.03 μg/ mL **(49)** IC_50_: 2.36 ± 0.08 μg/ mL **(50)** [61]
		Murrayamine-A **(51)** 1-O-Methylclausenolide **(52)**	IC_50_: 3.26 ± 0.14 μg/ mL on HeLa cells [61]
*Conyza sumatrensis* (Retz.) E. H. Walker Asteraceae Leaves	Eye diseases, paralysis, epilepsy, convulsion, tuberculosis, and asthma	Stigmast-5, 22-dien-3-O-*β*-*D*-glucopyranoside **(53)** 2, 3-dihydroxylpropyl hexacosanoate **(54)**	Chloroform sub-fraction 1 (GI_50_: 63.64 ± 1.33 μg/mL); sub-fraction 2 (GI_50_: 62.24 ± 0.18 μg/mL); sub-fraction 3 (GI_50_: 43.64 ± 0.7 μg/mL) and sub-fraction 4 (GI_50_: 79.89 ± 2.67 μg/mL) on MCF-7 cancer cells [62] Stigmast-5, 22-dien-3-O-*β*-*D*-glucopyranoside (GI_50_: 40.83 ± 0.1 μg/mL and 58.83 ± 11.2 μg/mL) for MCF-7 and NCI-H460 cancer cell lines, respectively 2, 3-dihydroxylpropyl hexacosanoate (GI_50_: 22.67 ± 1.33 μg/mL and 34 ± 5.6 μg/mL) for MCF-7 and NCI-H460 cancer cell lines, respectively [62]
*Eleusine indica* (L.) Gaertn Poaceae Roots	Diarrhoea, dysentery		Methanol fraction on RD cell line (IC_50_: 11.42 ± 1.01 μg/mL) [39] Hexane extract on HeLa (IC_50_: 466.3 ± 24.6 μg/mL) and A549 (IC_50_: 688.9 ± 60.1 μg/mL); Butanol extract on HeLa (IC_50_: 398.5 ± 24.9 μg/mL) and A549 (IC_50_: 753.7 ± 56.6 μg/mL) [63]
*Enterolobium cyclocarpum* (Jacq.) Griseb. Fabaceae Leaves	Inflammatory tumours and bronchitis		Methanol extract (IC50: 2.07 ± 1.30 μg/mL and 11.84 ± 1.18 μg/mL) on HeLa and MCF-7, respectively [64]
*Erythrophleum suaveolens* (Guill. & Perr.) Brenan Fabaceae	Emetic, respiratory problems	Erythrofordins D **(55)** Erythrofordins E **(56)**	Methanol extract on BT-549 (IC_50_: 0.55 ± 0.18 μg/mL), BT-20 (IC_50_: 0.50 ± 0.03 μg/mL), PC-3 (IC_50_: 1.30 ± 0.14 μg/mL), SW-480 (IC_50_: 0.80 ± 0.11 μg/mL) and Jurkat (IC_50_: 0.20 ± 0.05 μg/mL) [45]
*Fagara zanthoxyloides* Lam. Rutaceae Roots	Antimicrobial, genitourinary tract infection, sickle cell anemia, stomach disorders, sterility, and toothaches	Fagaronine **(57)**	Fagaronine **(57)** (IC_50_ = 3 × 10^–6^M) on K562 cancer cells [65] Aqueous extract on PC-3 (IC_50_: 25 ± 2.8 μg/mL), DU-145 (IC_50_: 25 ± 2.6 μg/mL), LNCaP (IC_50_: 39 ± 3.5 μg/mL), and CWR-22 (IC_50_: 44 ± 3.8 μg/mL) prostate cancer cell lines [66] Dichloromethane: methanol (1:1) extract percent growth inhibition on A549 (72%), PC-3 (71%), NCI-H322 (79%), and T47D (79%) cancer cells [67]
*Ficus sur* Forssk. Moraceae Leaves	Cancer		Methanol fraction on RD cell line (IC_50_: 19.23 ± 3.21 μg/mL) [39]
*Hoslundia opposita* Vahl. Labiatae	Abdominal pains, epilepsy, neurotic disorders [45]		Methanol extract on BT-549 (IC_50_: 76.4 ± 7.89 μg/mL), BT-20 (IC_50_: 56.1 ± 1.57 μg/mL) and PC-3 (IC_50_: 59.7 ± 8.11 μg/mL) [45]
*Hymemocardia acida* Tul. Hymenocardiaceae Stem bark	Used to treat hemorrhoids, chest pain, eye infections, migraine, skin diseases, abscesses, and tumors	Lupeol **(58)**	Methanol extract on H460 (IC50: 20.80 ± 6.10 μg/mL); MCF-7 (38.70 ± 0.80 μg/mL) and HCT116 (42.90 ± 0.20 μg/mL) [68]
*Jatropha curcas* Wall. Euphorbiaceae	Purgative, galactagogue, anticonvulsant ringworm, eczema, ulcer		Methanol extract on BT-549 (IC_50_: 21.3 ± 0.38 μg/mL) and BT-20 (IC_50_: 33.4 ± 0.70 μg/mL) [45]
*Justicia insularis* T. Anderson Acanthaceae Leaves	Digestive, weaning agent, laxative, and nutritional purposes	16-hydroxy-cleroda-3,13(14)*Z*-dien-15,16-olide **(59)** 16-oxo-cleroda-3,13(14)*E*-dien-15-oic acid **(60)**	16-hydroxy- cleroda-3,13(14)*Z*-dien-15,16-olide **(59)** on OVCAR-4 (IC_50_: 5.7 ± 0.3 μM); OVCAR-8 (IC_50_: 4.4 ± 0.2 μM) 16-oxo-cleroda-3,13(14)*E*-dien-15-oic acid on OVCAR-4 (IC_50_: 16.6 ± 2.8 μM) and OVCAR-8 **(60)** (IC_50_: 11.8 ± 0.5 μM) [69]
*Kigelia pinnata* (Lam.) Benth. Bignoniaceae Leaves, stem bark	Cancers, dysentery, syphilis, eczema, fungal infections, convulsions	(9*Z*, 12*Z*)-methyl octadeca-9,12-dienoate **(61)** Norviburtinal **(62)** and *β*-sitosterol **(33)**	Ethanol (IC_50_: 151.3 ± 0.9 ng/mL), Hexane (IC_50_: 143.4 ± 0.5 ng/mL), and methanol (IC_50_: 147.9 ± 1.3 ng/mL) fractions on RD human tissue cell line [70] Dichloromethane extract on G361 (IC_50_: 2.3 ± 0.1 µg/mL) cancer cells [71]
*Landolphia dulcis* var. barteri (Stapf) Pichon Apocynaceae Stem bark	Rheumatism, cough, kidney diseases, antibacterial		Methanol extract on BT-549 (IC_50_: 16.3 ± 4.31 μg/mL) [45]
*Lannea nigritana* (Sc. Elliot) Keay Anacardiaceae Leaves, stem bark, roots	None		Methanol extract on BT-549 (IC_50_: 48.2 ± 3.52 μg/mL) and Jurkat (IC_50_: 53.5 ± 0.35 μg/mL) [45]
*Lecaniodiscus cupanioides* Planch. ex Benth. Sapindaceae Leaves, stem	Cancer, laxative, galactogen, hepatomegaly, antibacterial, burns, wound, and cough	Phenolic constituents	Methanol fraction on RD cell line (IC_50_: 17.23 ± 1.98 μg/mL) [39]
	Breast cancer	3-O-[α-*L*-arabinofuranosyl- (1_→_3)-*α*-*L*-rhamnopyranosyl- (1→2)-*α*-*L*-arabinopyranosyl-]-hederagenin **(63)** 3-O-[*α*-*L*-arabinopyranosyl-(1→3)-*α*-*L*-rhamnopyranosyl (1→2)-*α*-*L*-arabinopyranosyl-]-hederagenin **(64)**	IC_50_ of compound **(63)** on H-116, A-549, and HT-29 cell lines: 5.0 μg/mL, 2.5 μg/mL, and 2.5 μg/mL, respectively IC_50_ of compound **(64)** on H-116, A-549, and HT-29 cell lines: 2.5 μg/mL, 5.0 μg/mL, and 5.0 μg/mL respectively [72]
*Macaranga barteri* Müll.Arg. Euphorbiaceae Leaves	Malaria, diabetes, bronchitis and cancer	3,5-dicaffeoylquinic acid **(65)** Acteoside **(66)** Kaempferol-7-O-glucoside **(67)** and Bastadin 11 **(68)**	Dichloromethane fraction on RD cell line (IC_50_: 0.22 ± 0.01 μg/mL) [39]
*Mimosa pudica* Linn. Mimosaceae Leaves	Kidney problems, fistula, cancer	Mimosine **(69)** Derivative of Myricetin: 2-(2’,6’-dimethyl-3’,4’,5’-alkyl or hydroxy alkyl substituted phenyl)-3-oxy-(alkyl or hydroxy alkyl)- 5,7-dihydroxy-chromen-4-one **(70–85)** R1, R2, R3 & R4 can be any of the four groups: 1: [2,4-dioxy bute-1,3-diene-1-ol] **(70–73)** 2: [7-oxy-2,4,6-heptatriene] **(74–77)** 3: [6-oxy-1,3,5-hexatriene] **(78–81)** 4: [1,3-butadiene] **(82–85)**	Methanol fraction on RD cell line (IC_50_: 2.03 ± 0.11 μg/mL) [39] Brine shrimp lethality assay of methanol extract (LC_50_: 282.35 µg/mL) [73] Brine shrimp lethality assay of methanol extract (LC_50_: 459.26 µg/mL) [74] Derivative of myricetin on A549 (76.67 ± 4.27 μg/mL) and K562 (287.63 ± 18.64 μg/mL) cell lines [75]
*Moringa oleifera* Lam. Moringaceae Leaves, seeds	Fever, asthma, cough, blood pressure, arthritis, diabetes, epilepsy, wound, and skin infection	Glucomoringin **(86)** Niazimicin **(87)** *β*-Sitosterol-3-O-*β*-d-glucopyranoside **(88)** 4(*α*-l-rhamnosyloxy)-benzyl isothiocyanate **(89)**	Aqueous extract on A549 cells (65% inhibition) [76] Aqueous extract on KB cell line (85% inhibition) [77] Dichloromethane extract on HepG2, Caco-2 and MCF-7 (IC_50_: 120.37 ± 2.55 μg/mL, 112.46 ± 3.74 μg/mL and 133.58 ± 2.47 µg/mL, respectively) [78] Aqueous extract on A549 cells (IC_50_: 166.7 μg/mL) [79] Ethyl acetate fraction on Hep-2 cell line (IC_50_: 12.5 ± 0.5 µg/mL) [80] Aqueous extract on Pac-1 (IC_50_: 1.1 mg/mL), COLO 357 (IC_50_: 1.8 mg/mL) and p34 (IC_50_: 1.5 mg/mL) cell lines [81] Aqueous extract on SW48 (IC_50_: 105.47 ± 23.50 μg/mL), SW480 (IC_50_: 200.26 ± 27.64 μg/mL) and HCT-15 (IC_50_: 266.67 ± 18.53 μg/mL) [82] Ethanol extract on SW48 (IC_50_: 102.40 ± 16.08 μg/mL), SW480 (IC_50_: 197.20 ± 32.52 μg/mL) and HCT15 (IC_50_: 264.83 ± 23.33 μg/mL) [82] Essential oil on MCF-7 (IC_50_: 226.1 µg/mL), HeLa (IC_50_: 422.8 µg/mL) and HepG2 (IC_50_: 751.9 µg/mL) [83] Glucomoringin **(86)** on H460 wild-type (IC_50_: 29.07 ± 0.76 µΜ), its subline H460 S5 (18.60 ± 6.65 µΜ), MCF7 π (IC_50_: 21.08 ± 5.67 µΜ), MCF-7 NEO (19.76 ± 4.38 µΜ) [84] Niazimicin **(87)** (IC_50_: 35.3 μg/mL), *β*-sitosterol-3-O-*β*-d-glucopyranoside **(88)** (IC_50_: 27.9 μg/mL), 4(α-l-rhamnosyloxy)-benzyl isothiocyanate **(89)** (IC_50_: 32.7 μg/mL) on induction of EBV-EA [85, 86]
*Nauclea latifolia* J. E. Smith Rubiaceae Stem bark	Cough, jaundice, stomach disorder, malaria fever, and cancer		Methanol fraction (100%), (IC_50_: 28.56 ± 0.34 μg/mL and 22.52 ± 0.34 μg/mL); Methanol: ethyl acetate fraction 1 (neutral fraction), (IC_50_: 3.23 ± 0.12 μg/mL and 13.15 ± 0.41 μg/mL); Fraction 2 (basic fraction), (IC_50_: 20.51 ± 0.28 μg/mL and 16.96 ± 0.44 μg/mL); fraction 3 (acidic fraction), (IC_50_: 61.98 ± 0.25 μg/mL and 69.60 ± 0.37 μg/mL); hexane/ethylacetate fraction (IC_50_: 16.70 ± 0.22 μg/mL and 8.97 ± 0.42 μg/mL) for MCF-7 and RD cancer cell lines, respectively [87]
*Olax mannii* Oliv. Olacaceae	Cancer and inflammation	Kaempferol 3-O-*α*-*L*-rhamnopyranoside **(90)**	Kaempferol 3-O-*α*-*L*-rhamnopyranoside **(90)** (IC50: 50 μM) against human K562 chronic myelogenous leukaemia cells [88]
*Parkia biglobosa* (Jacq.) Benth. Mimosaceae	Diarrhea and stomach aches, severe cough, wounds, dental caries, and sexually transmitted diseases		Petroleum ether (IC_50_: 5.4 ± 0.10 μg/mL) and ethyl acetate (IC_50_: 9.0 ± 0.34 μg/mL) fractions against SK-LU-1 lung carcinoma cell lines [89] Methanol extract on BT-549 (IC_50_: 100.0 ± 0.67 μg/mL), BT-20 (IC_50_: 125.0 ± 2.21 μg/mL), PC-3 (IC_50_: 56.1 ± 0.45 μg/mL) and SW-480 (IC_50_: 136.0 ± 0.81 μg/mL) [45]
*Peristrophe bicalyculata* (Retz.) Nees. Acanthaceae	Skin-related ailments, an antidote for snake poison, insect repellant		Partially purified fraction on KB (IC_50_: 3.50 ± 0.21 µg/mL) [90] Essential oil on MCF-7 (18.9 ± 5.7 µg/mL) and MDA-MB-468 (66.6 ± 36.8 µg/mL) [91] Chloroform (IC_50_: 6.21 ± 0.70 µg/mL), ethyl acetate (23.39 ± 3.92 µg/mL) and methanol fractions (22.43 ± 3.58 µg/mL) on HeLa cells; Chloroform (IC_50_: 1.98 ± 0.33 µg/mL), ethyl acetate (8.57 ± 1.91 µg/mL) and methanol fractions (28.24 ± 5.57 µg/mL) on MRC5-SV2 cancer cells [92]
*Pterocarpus santalinoides* L’Hér. ex DC. Fabaceae	Insecticidal, larvicidal		Methanol extract on BT-549 (IC_50_: 57.9 ± 0.35 μg/mL) and Jurkat (IC_50_: 10.2 ± 0.25 μg/mL) [45]
*Secamone afzelii* (Roem. & Schult.) K.Schum. Asclepiadaceae Leaves	Astringent, anthelminthic, cancer		Methanol fraction on RD cell line (IC_50_: 11.99 ± 2.01 μg/mL) [39]
*Sida acuta* Burm.f. Malvaceae	Malaria, ulcer, fever		Methanol extract on BT-549 (IC_50_: 10.3 ± 0.21 μg/mL), BT-20 (IC_50_: 41.1 ± 1.05 μg/mL), PC-3 (IC_50_: 37.1 ± 0.18 μg/mL) and Jurkat (IC_50_: 42.3 ± 0.79 μg/mL) [45]
*Solanum erianthum* D.Don Solanaceae Leaves	Treatment of sores and skin irritations	*α*-Terpinolene **(91)** *α*-Phellandrene **(92)** *p*-Cymene **(93)** *β*-Pinene **(94)**	Volatile oil on Hs 578T (IC_50_: 0.63 µg/mL) and PC-3 (IC_50_: 2.05 µg/mL) cells [93]
*Spondias mombin* Jacq. Anacardiaceae Leaves	Diarrhoea, dysentery, inflammation, antimalarial	*β*-Caryophyllene **(95)** *γ*-Cadinene **(96)**	Brine shrimp lethality assay of oils from fresh leaves (LC_50_: 0.01 μg/mL) and dried leaves (LC_50_: 4.78 μg/mL) [94] *Allium cepa* L. assay of aqueous extract (EC_50_: 1.3 mg/mL) [95]
*Terminalia ivorensis* A.Chev. Combretaceae Stem bark	Stomach ache, arthritis, constipation [96], cancer [39]		Methanol fraction on RD cell line (IC_50_: 13.42 ± 0.92 μg/mL) [39]
*Tetrapleura tetraptera* (Schumach. & Thonn.) Taub Leguminosae	Sickle cell		Methanol extract on BT-549 (IC_50_: 9.1 ± 1.40 μg/mL), BT-20 (IC_50_: 23.1 ± 7.05 μg/mL) and Jurkat (IC_50_: 37.5 ± 5.13 μg/mL) [45]
*Triclisia subcordata* Oliv. Menispermeaceae Roots	Antiulcer and antimicrobial	Cycleanine **(97)**	Ethanol extract on ovarian cancer (Ovcar-8) cells (IC_50_: 2.4 ± 0.5 μg/mL) [97]
*Vernonia amygdalina* Del. Asteraceae Leaves	Anti-malarial, anti-microbial, antidiabetic	Vernodalin **(98)**	Ethanol extract on MCF-7 (IC_50_: 56 µg/mL at 72 h) and MDA-MB-231 (IC_50_: 46 µg/mL at 72 h) [98]; MCF-7 (IC_50_: 5.68 ± 2 μg/mL) [99]
*Vitex doniana* Sweet Verbenaceae Bark, root	Gastroenteritis, diarrhea, antimicrobial		Methanol extract on BT-549 (IC_50_: 62.5 ± 0.23 μg/mL), BT-20 (IC_50_: 171.1 ± 1.33 μg/mL), SW-80 (IC_50_: 89.2 ± 6.65 μg/mL) and Jurkat (IC_50_: 84.0 ± 1.13 μg/mL) [45] Methanol extract on BT-549 (IC_50_: 44.9 ± 0.10 μg/mL), BT-20 (IC_50_: 152.3 ± 1.22 μg/mL), PC-3 (IC_50_: 177.3 ± 1.01 μg/mL), SW-80 (IC_50_: 45.6 ± 1.35 μg/mL) and Jurkat (IC_50_: 43.4 ± 0.64 μg/mL) [45]

RD: rhabdomyosarcoma; EACC: Ehrlich ascites carcinoma cell; EBV-EA: epstein-barr virus early antigen

## Cytotoxicity of Nigerian medicinal plants

The search for potential anticancer agents usually starts with *in vitro* inhibition assays on cancer cells to determine the effect of the extracts, fractions, or compounds on cell proliferation (cytostasis) or cell death (cytotoxicity). Plants tested for anticancer activities are chosen based on their traditional use or from databases [100]. Nigeria is among the top countries in Africa publishing research articles that provide increasing evidence for the potential of plants as inhibitors of tumorigenesis and associated inflammatory processes, signifying the importance of plants in cancer prevention and therapy [18, 39, 45, 46, 55, 70, 101]. Traditional healers in Nigeria claim to treat cancer successfully, but such claims require scientific validation. However, a majority of cancer patients in Nigeria prefer to seek cancer treatments from traditional healers, despite the lack, or weakness of efficacy proof, which could be attributed to several factors, including ignorance, poor education, poverty, and poor access to cancer medication. Unfortunately, as a result, most patients present at hospitals at advanced stages of the disease, when only chemotherapy and palliative care can be given [8, 9]. There is thus the need to gather information about Nigerian plants with potential anticancer activity from published articles and make the information available to scientists working in related areas so they could engage in more focused research that would discover lead compounds for the treatment of cancer and also clarify whether any of the herbal recipes has any merit of efficacy against cancer, which could help to inform the public about such remedies and thus safeguard public health.

Most inhibitory assays utilize IC_50_ (inhibitory concentration at 50% response), defined as the concentration of an extract or compound that causes a 50% reduction in the viability of cells in culture with respect to the negative control cells (cells not treated with the extract or compound) after a fixed incubation period [100, 102]. IC_50_ could also be defined in terms of the concentration that achieves 50% of the maximal (cytotoxic) response induced by a test agent. The Research and Training in Tropical Diseases (TDR, WHO—Tropical Diseases) uses IC_50_ to classify the extent of cytotoxicity as follows: not cytotoxic, IC_50_ is > 90 µg/mL; moderately cytotoxic, IC_50_ is between 2 µg/mL and 89 µg/mL; and cytotoxic if IC_50_ is < 2 µg/mL [100]. However, it is noteworthy that not all compounds or extracts are cytotoxic; some are cytostatic, that is, they are capable of inhibiting cell growth, through which they can prevent metastasis and, eventually over time, cause cell death [103]. This mechanistic difference should be considered during anticancer screening as highlighted in the present review.

### *Acanthospermum hispidum* (Asteraceae)

**Figure 4:**
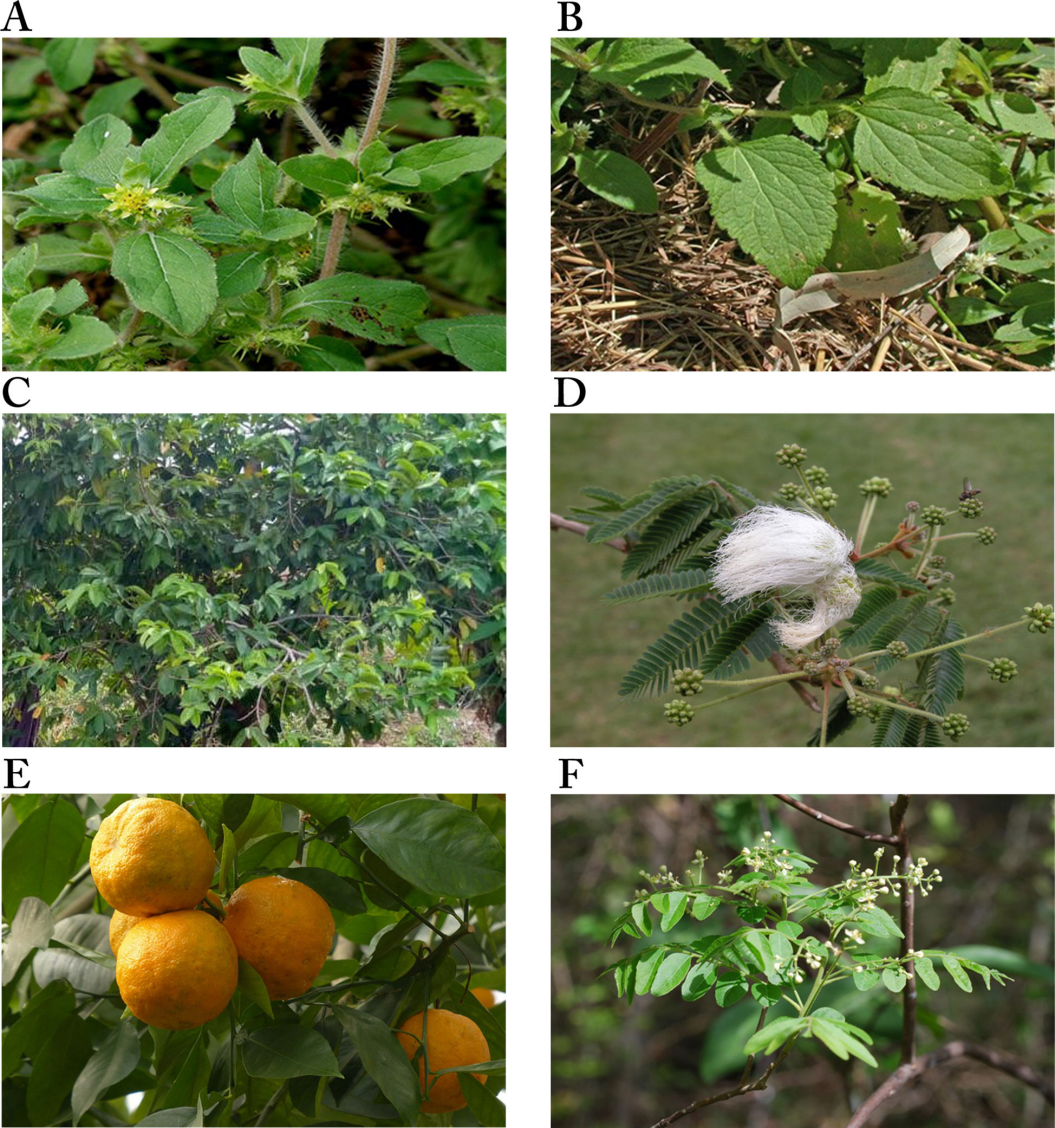
Images of some plants/plant parts. A. *Acanthospermum hispidum*; B. *Ageratum conyzoides*; C. *Annona muricata* tree; D. *Calliandra portoricensis*; E. *Citrus aurantium* L.; F. *Clausena anisata* showing inflorescence *Note.* (A) Adapted from “File: Acanthospermum hispidum W IMG 2208.jpg” by Garg JM (https://commons.wikimedia.org/wiki/File:Acanthospermum_hispidum_W_IMG_2208.jpg). CC BY 3.0; (B) reprinted with permission from “Australian Plant Image Index (APII) Photo No. dig 12854” by Richardson RG, Richardson FJ (https://www.anbg.gov.au/cgi-bin/photo?photo_class=dig&photo_no=12854). © R.G. & F.J. Richardson 2006; (C) Photograph taken in June, 2021 at Ibadan, Oyo State, Nigeria; (D) reprinted with permission from “Calliandra” by Goosen N (https://ngoosen.fotki.com/fabaceae/calliandra/calliandraportoricensis.html#media). © Nora Goosen. (E) adapted with permission from “Rind fruit bitter oranges-93679” by Hans (https://pixabay.com/photos/rind-fruit-bitter-oranges-93679/). CC0; (F) reprinted with permission from “Brunken, U., Schmidt, M., Dressler, S., Janssen, T., Thiombiano, A. & Zizka, G. 2008. West African plants - A Photo Guide. www.westafricanplants.senckenberg.de - Forschungsinstitut Senckenberg, Frankfurt/Main, Germany” by Porembski S (http://www.westafricanplants.senckenberg.de/root/index.php?page_id=14&id=369#image=24952). BY-NC.

*Acanthospermum hispidum* (Figure 4A), one of the six plants shown in Figure 4, is also called bristly starbur, goat’s head, or hispid starburr, and is an important plant in Nigeria because of its medicinal uses. It is native to South America but is now found in Africa and India [104]. In Nigeria, traditional medicine healers soak the leaves in palm wine for a day and squeeze them to obtain juice for the treatment of cancer [39, 101]. It has also been shown to possess antibacterial and antifungal properties [104]. The methanol extract of the plant was moderately cytotoxic against the human breast adenocarcinoma cell line (MCF-7), with IC_50_ values of 13.50 ± 1.00 μg/mL [38] and 19.92 ± 8.94 μg/mL [40]. It was also moderately cytotoxic against human large cell lung carcinoma (COR-L23, IC_50_: 8.87 ± 0.90 μg/mL), human amelanotic melanoma (C32, IC_50_: 13.54 ± 0.8 μg/mL) [38], rhabdomyosarcoma (RD, IC_50_: 19.65 ± 1.23 μg/mL), urinary bladder cancer (5637, IC_50_: 9.37 ± 1.98 μg/mL), and a lung cancer (A-427, IC_50_: 16.70 ± 2.32 μg/mL) cell lines [40]. The anticancer activity of the plant has been attributed to the compounds melampolides **(3)** and cis,cis-germacranolides **(4)**, both of which showed cytotoxic and *in vivo* anticancer activity [105].

### *Ageratum conyzoides* (Asteraceae)

*Ageratum conyzoides* (Figure 4B), commonly known as billygoat weed, is native to Central America but is now found in Africa and several Asian countries. The weed is known to interfere with the growth of crops, causing huge economic losses to farmers [106, 107]. In Nigeria, the Igedes in Benue State call it “ufu opioko” and “otogo”, while the Yorubas in Southwestern Nigeria call it “Imí esú” [43]. It is used for the treatment of fever, rheumatism, epilepsy, wounds, burns, cuts, and sores, as well as for throat infections [107]. The plant has been demonstrated to possess wound healing and antifungal properties [108]. The plant also demonstrated anticancer activity against some cancer cell lines. The ethyl acetate fraction of the plant was shown to be significantly cytotoxic against human non-small cell lung carcinoma (A-549, IC_50_: 0.68 μg/mL) and mouse leukemia (P-388, IC_50_: 0.0003 μg/mL) cell lines, and moderately cytotoxic against human gastric carcinoma (SGC-7901, IC_50_: 14.38 μg/mL) and human prostate carcinoma (DU-145, IC_50_: 9.90 μg/mL) cell lines. The IC_50_ values of the petroleum ether extract on SGC-7901, A549 and P-388 cancer cell lines were 13.77 μg/mL, 14.06 μg/mL and 0.71 μg/mL, respectively, while the ethanol extract was active against P-388 cancer cells with an IC_50_ value of 1.73 μg/mL, indicating the petroleum ether extract and the ethanol extract were significantly cytotoxic against P-388 cancer cells [43]. Thirteen compounds were isolated from the ethanol extract, with 7,3',5'-Tri-O-methyltricetin **(5)**, Precocene II **(6)**, 3,5,7,4'-tetrahydroxyflavone **(7)**, and 5,6,7,3',4',5'-hexamethoxyflavone **(8)** shown to inhibit the P-388 cancer cell line with IC_50_ values of 12.8 μM, 24.8 μM, 3.5 μM and 7.8 μM, respectively [44].

### Annona muricata (Annonaceae)

*Annona muricata* (Figure 4C) is widely distributed in Asia, Africa, and South America. It is commonly known as soursop (English), graviola (Portuguese), guanabana (Latin American Spanish), and other local indigenous names [109]. It is called “sharp sharp” by the Yorubas in Nigeria [101]. Its fruit, leaves, and other aerial parts are used as traditional medicines for the treatment of various diseases. The fruit is used for arthritis, diarrhea, dysentery, fever, malaria, parasites, etc., while diabetes, headaches, hypertension, and insomnia are treated with the leaves. Recent studies have demonstrated its anti-inflammatory and analgesic effects [110], and antiviral and antidiabetic activities [111]. The anticancer activities of the various parts of the plant have been extensively reported [48–52]. The acetogenin-enriched fraction and ethanol extract of the leaves were moderately cytotoxic against prostate cancer PC-3 cells with IC_50_ values of 57 μg/mL and 63 μg/mL, respectively [47]. The ethanol extract significantly inhibited human promyelocytic leukemia HL-60 cell line (IC_50_: 14 ± 2.4 μg/mL) [52] but was less cytotoxic against Ehrlich ascites carcinoma (EAC, IC_50_: 335.85 μg/mL), breast cancer (MDA-MB-231, IC_50_: 248.77 μg/mL) and SKBR3 (IC_50_: 202.3 μg/mL) cell lines [48]. The aqueous extract of the leaves was also less toxic on breast cancer cell lines MCF-7 (IC_50_: 221.67 ± 1.67 μg/mL), MDA-MB-231 (IC_50_: 350 ± 5.77 μg/mL), and 4T1 (IC_50_: 251.67 ± 6.01 μg/mL) [49], while the ethyl acetate extract was moderately toxic against breast cancer cell lines MCF-7 (IC_50_: 6.39 ± 0.43 μg/mL) and MDA-MB-231 (IC_50_: 11.36 ± 0.67 μg/mL), human lung cancer cells (A549, IC_50_: 5.09 ± 0.41 μg/mL), human hepatoma cells (HepG2, IC_50_: 9.3 ± 0.91 μg/mL), human hepatic cells (WRL-68, IC_50_: 47.10 ± 1.23 μg/mL) [50], and human colon cancer cells HCT-116 (IC_50_: 8.98 ± 1.24 μg/mL) and HT-29 (IC_50_: 11.43 ± 1.87 μg/mL) [51]. The ethanol extract of the roots of *A. muricata* was more potent against human promyelocytic leukemia (HL-60) than extracts from the twigs, with IC_50_ values of 9 ± 0.8 μg/mL and 49 ± 3.2 μg/mL, respectively [52]. The leaves, roots, seeds, and twigs of *Annona muricata* are rich in flavonoids, isoquinoline alkaloids, and annonaceous acetogenins, with over 200 compounds isolated [112]. Many of the compounds have exhibited cytotoxic activity against several cancer cell lines, e.g., Muricin J **(13)**, K **(14)** and L **(15)** from the fruits, Annomuricin A **(16)**, B **(17)**, C **(18)** and E **(19)**, Annohexocin **(20)**, Muricapentocin **(21)**, and Annopentocin A **(22)**, B **(23)** and C **(24)** (Table 1) from the leaves, all annonaceous acetogenins [109].

### *Calliandra portoricensis* Jacq. Benth (Leguminosae)

*C. portoricensis* (Figure 4D), also called powder-puff or snowflake acacia, belongs to the Leguminosae family. It is native to Central America, most precisely Mexico, Panama, and West Indies, but grows in West African countries like Nigeria and Ghana [56]. It is one of the medicinal plants used in the treatment of arthritis [113], convulsion, breast engorgement [114], malaria, stomach ulcers, and cancer [39, 101] in Southwestern Nigeria where it is called “tude” [39, 114]. Pharmacological studies have demonstrated that the root and leaf extracts of the plant possess analgesic, anti-ulcerogenic, and anticonvulsant properties. The root is used to treat gonorrhea after mixing with pepper. It is added to snuff to promote sneezing, relief of headaches, and ophthalmic preparations [56]. Studies by Ogbole et al. [39] demonstrated that the ethyl acetate extract of the root bark of *C. portoricensis* was significantly cytotoxic against the RD cell line, with an IC_50_ of 0.82 ± 0.08 μg/mL. On the other hand, the methanol fraction was moderately cytotoxic against prostate cancer cell lines LNCaP (IC_50_: 2.4  ± 0.2 µg/mL) and DU-145 (IC_50_: 3.3  ±  0.2 µg/mL), and lung cancer cells (IC_50_: 3.6  ±  0.2 µg/mL) [57], while Adaramoye et al. [56] reported it was less cytotoxic against PC-3 (IC_50_: 31.32 ± 4.61%) and LNCaP (IC_50_: 11.76 ± 1.0%). Brine shrimp lethality assay carried out to determine the lethal concentration of the aqueous and methanol extracts of *C. portoricensis* root bark showed that the aqueous extract was significantly cytotoxic to brine shrimp, causing 96.67%, 86.67%, and 80% mortality at 1,000 ppm, 100 ppm, and 10 ppm, respectively, while the methanol extract caused 100%, 83.30%, and 90% mortality at the same concentrations. Both extracts had LC_50_ of 0.18% and 0.88% for aqueous and methanol extracts, respectively, indicating they are significantly cytotoxic [115]. Fractions of the plant have also been shown to possess antioxidant and anti-inflammatory activities [116].

The anticancer properties of the plant have been attributed to the alteration of the Bax/Bcl-2 ratio and growth arrest in prostate LNCaP cells. There was a 3-fold decrease in the expression of Bcl-2 and a 4-fold increase in Bax levels when LNCaP cells were treated with 10 μg/mL methanol extract of the roots. Also, the extract induced cytochrome C release by 4.2 folds and decreased fluorescence intensity ratio by 3.5 folds at the same concentration relative to the control, causing growth arrest at the S phase [57].

High Performance Liquid Chromatographic (HPLC) analysis of the methanol extract of *C. portoricensis* showed that the major components were Neurolenin B **(37)**, Nigrosporolide **(38)**, and *trans*-geranic acid **(39)** [39].

### *Citrus aurantium* L. (Rutaceae)

The positive effects of citrus plants on human health were known centuries before researchers began to unravel their beneficial biological activities. Several studies have been carried out to establish these biological activities and identify the bioactive components present in the different parts of citrus fruits, just as is done with other plants known to possess some health benefits [117, 118].

*C. aurantium* (Figure 4E), commonly called sour or bitter orange, belongs to the family Rutaceae. It is used in many cultural cuisines and in juice production because of its nutrients, flavor, and intrinsic attributes [58]. It is among the species that have been used for medicinal purposes on account of their bioactive compounds such as phenolics, flavonoids, essential oils, and vitamins. The antibacterial, anticancer, antidiabetic, antifungal, anti-hypertensive, anti-inflammatory, anti-lipidemic, and antioxidant properties of the fruit peel, flowers, and leaves have been reported [59]. Also, it’s been reported to protect the heart, liver, and bone, and prevent urinary diseases [58, 118]. It is traditionally used to treat cancer, diabetes, malaria, typhoid, and worm infestations, and to manage anorexia and obesity [101].

The anticancer properties of *C. aurantium* have been reported in several studies. The dichloromethane fraction of the bark was shown to be moderately cytotoxic against human lung (A549, IC_50_: 3.88 µg/mL), breast (MCF-7, IC_50_: 5.12 ± 0.54 µg/mL), prostate (PC-3, IC_50_: 4.72 ± 0.23 µg/mL) and human liver hepatocellular (HepG2, IC_50_: 5.73 µg/mL) carcinoma cells, while the methanol extract of the root bark was less cytotoxic: A549 (IC_50_: 88.9 ± 1.23 µg/mL), HepG2 (IC_50_: 92.7 ± 4.11 µg/mL), MCF-7 (IC_50_: 90.6 ± 4.54 µg/mL) and PC-3 (IC_50_: 78.2 ± 2.14 µg/mL) [101]. However, the methanol extract of the bloom was moderately cytotoxic against the human cancer cell line MDA-MB-231 (IC_50_: 49.74 ± 0.75 µg/mL), while its IC_50_ values against human colon adenocarcinoma (HT-29, IC_50_: 96.23 ± 0.75 µg/mL) and another human cancer cell line (MCF-7, IC_50_: 152.34 ± 0.75 µg/mL) [115] showed it was less cytotoxic [100].

**Figure 5:**
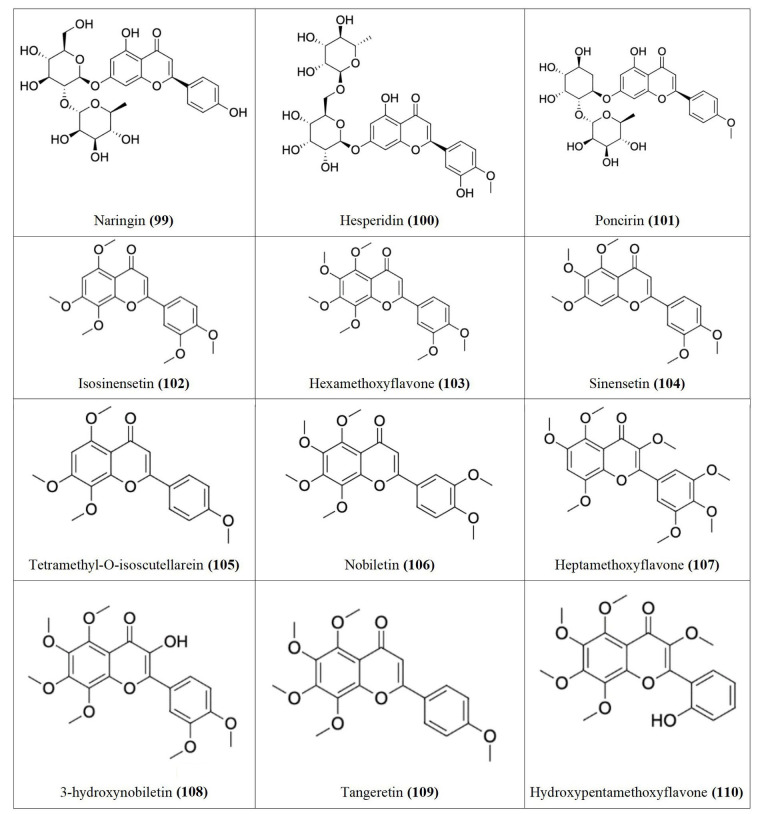
Flavonoids **99–110** isolated from *Citrus aurantium*

The anti-proliferative activity of flavonoids isolated from *C. aurantium* against human gastric cancer cells (AGS) showed that flavonoids inhibited cell viability in a concentration-dependent manner, with an IC_50_ of 99 µg/mL [58]. In another report, flavonoids were shown to inhibit human lung carcinoma cells (A549) with an IC_50_ of 230 µg/mL [59] and human hepatocarcinoma cells (HepG2) with an IC_50_ of 75 µg/mL [60]. Flavonoids isolated from *C. aurantium* include flavanones [Naringin **(99)**, Hesperidin **(100)**, and Poncirin **(101)**] and flavones [Isosinensetin **(102)**, Hexamethoxyflavone **(103)**, Sinensetin **(104)**, Tetramethyl-O-isoscutellarein **(105)**, Nobiletin **(106)**, Heptamethoxyflavone **(107)**, 3-hydroxynobiletin **(108)**, Tangeretin **(109)** and Hydroxypentamethoxyflavone **(110)**], with Naringin **(99)**, Hesperidin **(100)**, and Nobiletin **(106)** being the major ones (Figure 5) [58–60].

Flavonoids have been reported to induce G2/M arrest and thus induce apoptosis by regulating cell cycle-dependent and pro-apoptotic proteins, thus activating caspase 3, up-regulating Bax/Bcl-xL, caspase 3 activity, and cleaved poly ADP-ribose polymerase (PARP); and down-regulating pro-caspases (caspase-3, -6, -8 and -9) proteins, suggesting *C. aurantium* may be beneficial for the treatment of cancer [58–60].

Also, alkaloids isolated from the methanol extract of the root bark demonstrated potent to moderate cytotoxicity (IC_50_: 12.65–50.74 µM) against cancer cells and were at least four times more selective towards the carcinoma cells than the normal human immortalized prostate (PNT2) cells [119].

### *Clausena anisata* (Rutaceae)

*C. anisata* (Figure 4F) is native to Africa, mainly West Africa and North Africa. It is commonly known as “Horse wood”. It is called “Atabari Obuko” by the Yorubas in Southwestern Nigeria where it is used to treat malaria, cancer, and gut disturbance [101]. For gut disturbance, it is mixed with *Afraegle paniculata* and *Azadirachtha indica*, while a combination of *C. anisata* and *Azadirachtha indica* leaves is used to treat malaria [120]. The leaves of *C. anisata*, *Ageratum conyzoides*, *Momordica charantia,* and *Uvaria chamae* are boiled together in an earthen vessel containing pap water for the treatment of cancer [101]. It is also used by traditional healers in Tanzania against oral candidiasis and fungal infections, epilepsy, and as an anticonvulsant. A decoction from the root is used in children to control convulsions and as a tonic for pregnant women [61]. Researchers have demonstrated the antioxidant, antidiabetic, antibacterial, anti-inflammatory, antiviral, and cytotoxic properties of the leaves, roots, and stem bark of *C. anisata* [120].

Ogbole et al. [39] demonstrated that the methanol fraction of *C. anisata* leaves was moderately cytotoxic against human RD cells (IC_50_: 8.83 ± 0.59 μg/mL), and its LC_50_ value from the brine shrimp lethality assay (318.2  ±  8.12 μg/mL) indicates the plant contains bioactive secondary metabolites. In another study, Tatsimo et al. [61] reported the cytotoxicity of alkaloids isolated from the leaves and stem bark of *C. anisata* against HeLa cells. From their results, the alkaloids 3-(1,1-dimethyl allyl) xanthyletin **(46)**, Gravelliferone **(47)**, and 7-[(*E*)-7-hydroxy-3,7-dimethylocta-2,5-dienyloxyl]-coumarin **(49)** were significantly cytotoxic against HeLa cells, with the following IC_50_ values: 1.14 ± 0.16 μg/mL, 1.81 ± 0.09 μg/mL and 1.27 ± 0.03 μg/mL, respectively, while Excavatin D **(48)** and Phellopterin **(50)** were moderately cytotoxic (IC_50_: 2.36 ± 0.08 μg/mL and 3.26 ± 0.14 μg/mL, respectively).

### *Erythrophleum suaveolens* (Fabaceae)

**Figure 6:**
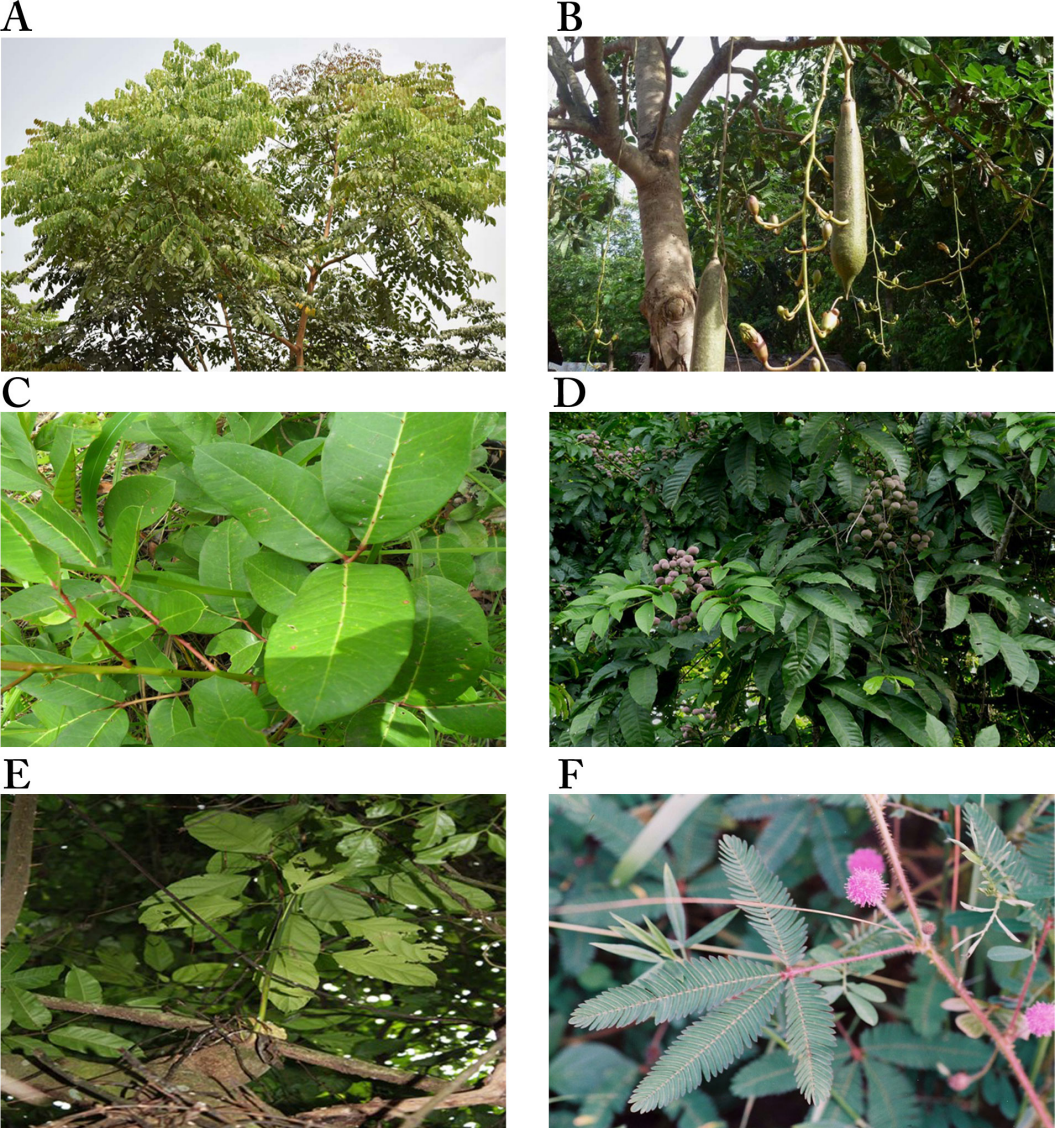
Images of some plants/plant parts. A. *Erythrophleum suaveolens*; B. *Kigelia pinnata* (or *Kigelia africana*) flowering and fruiting; C. *Fagara zanthoxyloides* (*Zanthoxylum zanthoxyloides*); D. fruiting branches of *Lecaniodiscus cupanioides;* E. *Macaranga barteri* tree; F. a stalked flowering head of *Mimosa púdica* *Note.* (A) Reprinted with permission from “*ERYTHROPHLEUM SUAVEOLENS*” by Olubodun O (https://forestcenter.iita.org/index.php/2019/06/21/erythrophleum-suaveolens/). CC BY-NC 2.0.; (B) adapted with permission from “*KIGELIA AFRICANA*” by Bown D (https://forestcenter.iita.org/index.php/2019/07/23/kigelia-africana/). CC BY-NC 2.0.; (C) adapted with permission from “Brunken, U., Schmidt, M., Dressler, S., Janssen, T., Thiombiano, A. & Zizka, G. 2008. West African plants - A Photo Guide. www.westafricanplants.senckenberg.de - Forschungsinstitut Senckenberg, Frankfurt/Main, Germany” by Ouédraogo O (http://www.westafricanplants.senckenberg.de/root/index.php?page_id=14&id=1681#image=7870). CC BY-NC. (D). reprinted with permission from “*LECANIODISCUS CUPANIOIDES*” by Bown D (https://www.flickr.com/photos/iita-media-library/6240362196/in/photostream/). CC BY-NC 2.0; (E) reprinted with permission from “Brunken, U., Schmidt, M., Dressler, S., Janssen, T., Thiombiano, A. & Zizka, G. 2008. West African plants - A Photo Guide. www.westafricanplants.senckenberg.de - Forschungsinstitut Senckenberg, Frankfurt/Main, Germany” by Schmidt M (http://www.westafricanplants.senckenberg.de/root/index.php?page_id=14&id=2476#image=7380). CC BY-NC. (F) reprinted with permission from “Brunken, U., Schmidt, M., Dressler, S., Janssen, T., Thiombiano, A. & Zizka, G. 2008. West African plants - A Photo Guide. www.westafricanplants.senckenberg.de - Forschungsinstitut Senckenberg, Frankfurt/Main, Germany” by Latham P. (http://www.westafricanplants.senckenberg.de/root/index.php?page_id=14&id=2528#image=41752). CC BY-NC.

*E. suaveolens* (Figure 6A) is a low-branching perennial tree about 30 m high, with a dense crown. It is native to Africa but also found in Asia [121]. It belongs to the Fabaceae family and is commonly called “Sasswood” [121] or “red water tree” [122]. In Nigeria, it is called “Obo” in Yoruba, “Nyi/Ihi” in Igbo, and “Gwaska” by the Hausas [121]. The plant is believed to be poisonous hence, it is used traditionally to scare offenders during investigations (based on a traditional belief system for identifying an offender in a crime) [123]. The stem is used together with *Belina acuminata* in Cameroon for the treatment of dermatitis, convulsion, and inflammation due to venom intoxication, as well as the treatment of cardiac diseases, headaches, and migraines [124]. In Nigeria, the stem bark is mixed with *Allium ascalonicum* and the root of *Tetracera alnifolia* to produce local soap and cream for the treatment of breast cancer [121]. Studies have demonstrated the anti-inflammatory, analgesic [124], and antibacterial [122] properties of the stem bark of *E. suaveolens*.

Fadeyi et al. [45] reported that *E. suaveolens* exhibited cytotoxic activity against several cancer cell lines compared to other medicinal plants tested. The IC_50_ values of the methanol extract of the plant against all cancer cell lines tested (BT-549, BT-20, PC-3, SW-480 and Jurkat cell lines) were 0.55 ± 0.18 μg/mL, 0.50 ± 0.03 μg/mL, 1.30 ± 0.14 μg/mL, 0.80 ± 0.11 μg/mL and 0.20 ± 0.05 μg/mL, respectively [45], which are significantly lower than 2 μg/mL, indicating the plant is significantly cytotoxic and possesses anticancer potential [100].

The plant has been shown to contain several medicinal compounds [125], including phenolic compounds like catechin, gallic acid, and pyrogallol [126]. Two cassaine-type diterpenoids isolated from *E. suaveolens*, Erythrofordins D **(55)** and E **(56)**, were found to be cytotoxic and responsible for the anticancer activity of the crude extract of the plant [125]. The LC_50_ of the compounds was 26.92 μM and 11.48 μM for Erythrofordins D **(55)** and E **(56)**, respectively. In addition, the compounds showed cardiotoxic effects at very low concentrations, hence the need for further *in vivo* studies focusing on their safety for the treatment of cancer [125].

### *Kigelia pinnata* (Bignoniaceae)

*K. pinnata* (Figure 6B), of the family Bignoniaceae, is native to Africa and widely distributed in the South, Central and West regions. It is commonly called “the sausage tree” or “cucumber” in English due to its large fruits [127, 128]. The Afrikaans call it “worsboom”, and Yorubas from Southwestern Nigeria call it “pandoro” [129]. It is a multipurpose plant with a variety of medicinal uses. The leaves are used to treat gastrointestinal ailments, stem bark for the treatment of dysentery, syphilis, eczema, fungal infections, convulsions, and as an antidote for snakebite [130]. The fruits are baked to ferment beer and prevent face blemishes, as purgative, and to increase milk flow in lactating mothers [127]. The plant also has some non-medicinal uses, as its leaves and fruits are eaten by animals. It is also used for funeral rites and to “hunt witches” in some parts of Africa [128].

Studies have demonstrated the antidiarrheal, antileprotic, antimalarial, anti-inflammatory, antimicrobial, and anticancer activities of the plant [127, 129]. The fruit and stem bark of *K. pinnata* have been shown to be cytotoxic *in vivo* and against some cancer cell lines [130]. The dichloromethane extract of the stem bark was cytotoxic against the human melanoma cell line (G361), with an IC_50_ of 2.3 ± 0.1 µg/mL. On further purification, three compounds, Norviburtinal **(62)** and *β*-sitosterol **(33)** were isolated from the extract, and Norviburtinal **(62)**, which exhibited the highest cytotoxic effect (IC_50_ of 3.25 ± 0.97 µg/mL), was considered responsible for the cytotoxic activity of the extract [71] In another study, Atolani et al. [70] reported that the ethanol, hexane, and methanol extracts of the leaves of *K. pinnata* showed significant cytotoxicity (IC_50_) of 151.3 ± 0.9 ng/mL, 143.4 ± 0.5 ng/mL and 147.9 ± 1.3 ng/mL, respectively, against RD human cancer cell line, each of which was more potent than the cytotoxicity of the reference standard, cyclophosphamide (IC_50_: 165.6 ± 1.0 ng/mL). They attributed the extremely potent cytotoxicity to the compound (9Z, 12Z)-methyl octadeca-9,12-dienoate **(61)** (IC_50_: 153.3 ± 0.1 ng/mL) isolated from the leaves.

### *Fagara zanthoxyloides* Lam. (Rutaceae)

There are over 549 species belonging to the *Zanthoxylum* (Rutaceae) species worldwide, with 35 reported in Africa [131] and 11 identified in Nigeria [132]. These eleven *Zanthoxylum* species are *Z. bouetense, Z. buesgenii, Z. dinklagei, Z. gillettii, Z. lemairie, Z. leprieurii, Z. rubescens, Z. tessmannii, Z. thomensee, Z. viride* and Z. *zanthoxyloides*, all of which are similar in characteristics and are identified as trees, erect shrubs or small trees, straggling or scandent shrubs, or as a forest liane [132]. The similar morphological characteristics of *Fagara* and the *Zanthoxylum* genera cause confusion; however, the opposite single/double perianth is used to distinguish them [133].

The root bark of *F. zanthoxyloides*, also known as *Z. zanthoxyloides* (Figure 6C), is used traditionally for its anti-fungal and anti-malarial properties, and to treat sickle cell anemia. In Nigeria, it is used by traditional healers for the treatment of a wide range of disorders, including toothache, urinary and venereal diseases, rheumatism, and lumbago [66, 131]. Its antimicrobial, antihypertensive, anti-sickling, antiproliferative, and anti-inflammatory properties have been demonstrated [67, 131, 132, 134].

*F*. *zanthoxyloides* is indigenous to and widely used in West Africa, where its roots and slim stem are used as chewing sticks for teeth cleaning [66, 67]. These chewing sticks are usually pencil-sized sticks of about 6 inches or 15 mm long, used frequently during the day to prevent different types of oral disease, including periodontal disease and dental caries [67, 134]. It is interesting to know that a significant correlation between periodontal disease and Vitamin D deficiency and the incidence of several cancers, including oral and breast cancers, has been established [135]. At the same time, extracts from the leaves, fruits, stems, and root bark have been shown to possess antiproliferative activities [67, 131].

Fagaronine **(57)**, an alkaloid isolated from the roots of *F*. *zanthoxyloides,* exhibited potent antitumor effect against P388 and L1210 murine leukemia cells [136], and K562 cancer cells (IC_50_: 3 × 10^–6^M) [65]. In another study, Kassim et al. [66] reported the cytotoxic activity of the root bark of *F. zanthoxyloides* against two androgen-independent prostate cancer cell lines: PC-3 (IC_50_: 25 ± 2.8 μg/mL) and DU-145 (IC_50_: 25 ± 2.6 μg/mL); and two androgen-dependent prostate cancer cell lines: LNCaP (IC_50_: 39 ± 3.5 μg/mL) and CWR-22 (IC_50_: 44 ± 3.8 μg/mL); and suggested the plant could serve as a potential chemotherapeutic agent for the treatment of prostate cancer. The dichloromethane: methanol (1:1) extract of the roots resulted in 72%, 71%, 79%, and 79% growth inhibition against A549, PC-3, NCI-H322, and T47D cancer cells using the sulforhodamine B (SRB) assay [67]. Other synthetic analogues of fagaronine have been developed and their anticancer activities established. Fagaronine **(57)** and its analogs have been shown to be DNA-intercalating agents and inhibitors of topoisomerases I and II [137].

### *Lecaniodiscus cupanioides* (Sapindaceae)

*Lecaniodiscus cupanioides* (Figure 6D), from the family Sapindaceae, is a tropical plant widely distributed in Africa and Asia. In Nigeria, it is called “kafi nama zaki” by the Hausas, “ukpo” by the Igbos, and “arika” by the Yorubas, and widely used for the treatment of wounds, boils, burns, toothache, fever, and hepatomegaly [138]. The leaves are used as spice for postnatal well-being [139], and the root decoction as a chemotherapeutic agent against rheumatism [140]. Aqueous root extract of the plant has been reported to possess antimalarial [138], central nervous system (CNS) depressant, analgesic, and hepatotoxic properties [141, 142], while the methanol extract has been demonstrated to possess antimicrobial and antioxidant activities and to be cytotoxic [143]. In a study to determine the *in vitro* cytotoxic activity of medicinal plants from Nigeria, Ogbole et al. [39] reported that the methanol fraction of the plant was moderately cytotoxic against the RD cell line (IC_50_: 17.23 ± 1.98 μg/mL). Two triterpenoid saponins: 3-O-[*α*-*L*-arabinofuranosyl- (1→3)-*α*-*L*-rhamnopyranosyl-(1→2)-*α*-*L*-arabinopyranosyl-]-hederagenin **(63)** and 3-O-[*α*-*L*-arabinopyranosyl-(1→3)-*α*-*L*-rhamnopyranosyl (1→2)-*α*-*L*-arabinopyranosyl-]-hederagenin **(64)**, isolated from the stem of the plant, show cytotoxic against human colon carcinoma H-116, human lung carcinoma A-549 and human colon carcinoma HT-29 cell lines. The IC_50_ values of compound **(63)** against H-116, A-549, and HT-29 cell lines were 5.0 μg/mL, 2.5 μg/mL, and 2.5 μg/mL, respectively, and for compound **(64)** against the same cells were 5.0 μg/mL, 5.0 μg/mL, and 2.5 μg/mL, respectively [72].

### *Macaranga barteri* (Euphorbiaceae)

*M. barteri* (Figure 6E) is a shrub or tree commonly found in Guinea, Southern Nigeria, and Equatorial Guinea. In folk medicine, the plant is used as a vermifuge and a febrifuge, and to treat wounds and swellings, cough, and bronchitis. Previous studies on the leaves have reported it is a rich source of flavonoids, terpenes, tannins, coumarins [144], and trace amounts of alkaloids, although none in the bark [145]. The bark and leaf, either powdered or in decoction, are used as a vermifuge [145]. The leaves are used for treating gonorrhea in Sierra Leone and as an anti-anaemic tonic in Cote d’Ivoire. In Southwest Nigeria, it is mixed with different leaves for the treatment of cancer [119, 121] Some people combine its stem bark with the leaves of *Vernonia amygdalina*, *Pseudocedrela kotschyi*, and *Khaya ivorensis* by boiling with pap water, before the addition of honey; for others, the root is mixed with *Uvaria afzelii*, stem bark of *Uvaria chamae*, *Securidaca longipedunculata* and the leaves of *Hoslundia opposita* and cooked with water in an earthen vessel to treat various cancers [101, 119]. Studies on the pharmacological activities of the plant indicate its antioxidant [141], antidiabetic [146], antimicrobial [147], and anti-inflammatory [148] potential. Gas chromatography-mass spectrometry (GC-MS) analysis of *M. barteri* revealed neophytadine (6.01%), *trans*-caryophyllene (5.03%), methyl hexadecanoate (4.00%) and phytol (2.32%) as major constituents of the methanol fraction, indicating the leaves as a source of bioactive constituents with therapeutic potentials [146].

Although there are reports of the anticancer properties of several plants of the genus *Macaranga* [144], there is a paucity of information on the anticancer effects of *M. barteri.* A study by Ogbole et al. [39] demonstrated that the dichloromethane extract of the leaves was significantly cytotoxic against RD cancer cell line (IC_50_: 0.22 ± 0.01 μg/mL), with a comparable lethal activity on the brine shrimp and a high degree of selectivity against cancer cells. They also established that 3,5-dicaffeoylquinic acid **(65)**, Acteoside **(66)**, Kampferol-7-*O*-glucoside **(67)**, and Bastadin 11 **(68)** were the major components of the extract.

### *Mimosa pudica* (Mimosaceae)

*Mimosa pudica* (Figure 6F) is a weed easily dismissed as invasive, and commonly known as sleeping grass, sensitive plant, humble plant, shy plant, and touch-me-not. It is of the Mimosaceae family with thigmonastic and nyctinastic movements that make it an ornamental plant [149]. It is a popular plant used by folk healers to treat several diseases such as biliary disease, bilious fevers, piles, jaundice, leprosy, cancer, diabetes, hepatitis, obesity, and urinary infections [149, 150]. It is called “Patanmo” by the Yorubas in Southwestern Nigeria, where it is used to treat cancer [100]. Its potent antioxidant, anti-inflammatory, antimicrobial [151], and antiviral [152] properties have been reported. The plant has also been reported to possess antinociceptive, antidepressant [153], hypolipidaemic [154], hepatoprotective [155], wound-healing [156], and anticonvulsant [157] effects, among other pharmacological properties [149, 158], all of which have been attributed to the phytochemical constituents of the plant.

**Figure 7:**
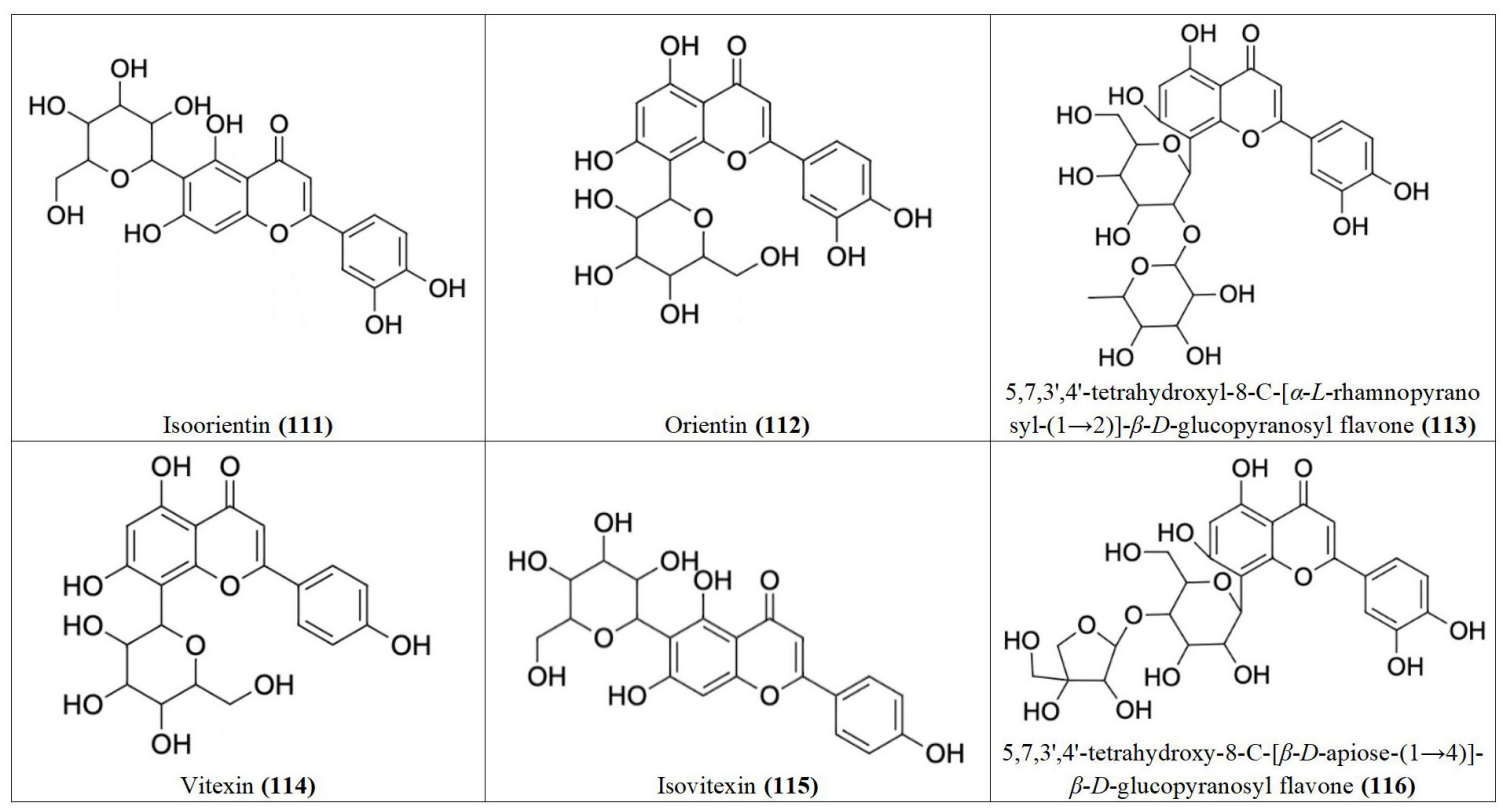
Flavones **111–116** isolated from *Mimosa pudica*

The methanol fraction of the plant has been shown to be significantly cytotoxic against the RD cell line (IC_50_: 2.03 ± 0.11 μg/mL) [39], while brine shrimp lethality assay of the extract gave LC_50_ values of 282.3495 µg/mL [73] and 459.25 µg/mL [74], suggesting the plant may be a potential source of anticancer compounds. Several compounds with anticancer activity have been isolated from *M. pudica.* Jose et al. [75] isolated a novel anticancer flavonoid, 2-(2’,6’-dimethyl-3’,4’,5’-alkyl or hydroxy alkyl substituted phenyl)-3-oxy-(alkyl or hydroxy alkyl)-5,7-dihydroxy-chromen-4-one **(70)**, from *M. pudica* with significant concentration-dependent cytotoxic effects against A549 (IC_50_: 76.67 ± 4.27 μg/mL) and human erythroleukemic cell line (K562, IC_50_: 287.63 ± 18.64 μg/mL). The compound also exhibited *in vivo* anticancer activity by preventing the proliferation of Dalton’s Ascites Lymphoma (DAL) in the peritoneal region of Swiss albino mice due to its cytotoxic activity and it prolonged the life span of the mice. Six other flavonoids: Isoorientin **(111)**, Orientin **(112)**, 5,7,3’,4’-tetrahydroxyl-8-C-[*α*-*L*-rhamnopyranosyl-(1→2)]-*β*-*D*-glucopyranosyl flavone **(113)**, Vitexin **(114)**, Isovitexin **(115)** and 5,7,3’,4’-teteahydroxy-8-C-[*β*-*D*-apiose-(1→4)]-*β*-*D*-glucopyranosyl flavone **(116)** (Figure 7), have also been isolated from *M. pudica* and shown to possess antiproliferative activities against MCF-7, embryonic human chorionic (JAR) and neuroblastoma (N-2A) cancer cell lines [159].

### *Moringa oleifera* Lam (Moringaceae)

**Figure 8:**
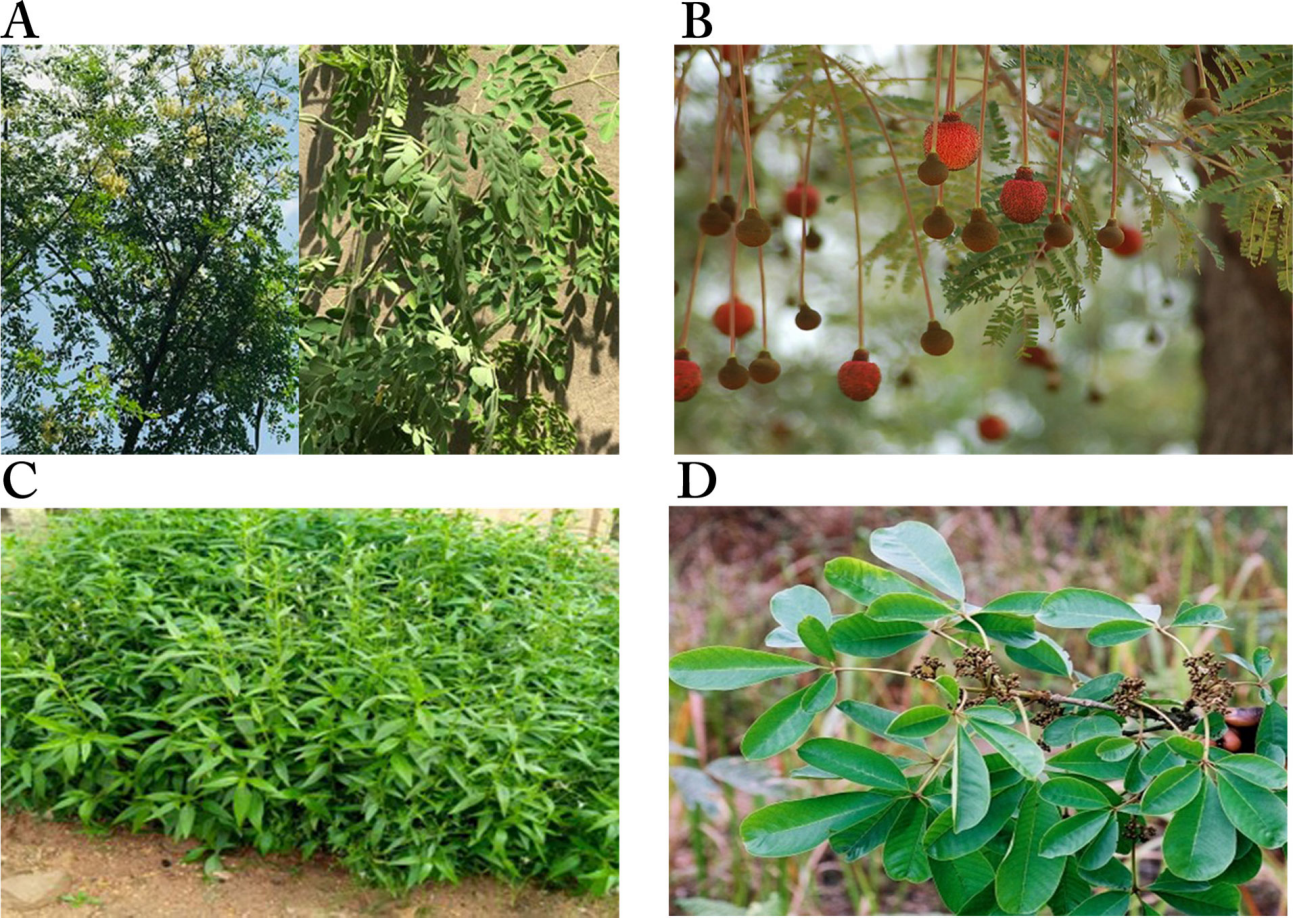
Images of plants/plant parts. A. Tree and leaves of *Moringa oleifera*; B. *Parkia biglobosa* leaves and inflorescences. C. shrubs of *Peristrophe bicalyculata*; D. the leaves of *Vitex doniana* *Note.* (A) Photograph taken in June 2021 at Rijiyan Zaki Area, Kano State, Nigeria; (B) Reprinted with permission from “*Parkia biglobosa*” by iNaturalist (https://www.inaturalist.org/taxa/133528-Parkia-biglobosa). CC-BY; (C) Photograph taken in June 2021 at Ibadan, Oyo State, Nigeria; (D) adapted with permission from “West African plants—A Photo Guide” by Latham P (http://www.westafricanplants.senckenberg.de/root/index.php?page_id=14&id=1661#image=56072). CC BY-NC.

*M. oleifera* or “drumstick” (Figure 8A), one of the four plants shown in Figure 8, is a member of the Moringaceae family used by the ancient Romans, Greeks, and Egyptians, but has been naturalized to the sub-Himalayan regions such as India, and widely distributed in Asia, Latin America, and Africa [76, 79]. It is referred to as the “tree of life” or “miracle vegetable” because of its multipurpose uses as a source of food and medicine for humans and animals, forestry products, and a source of fertilizer [76]. All parts of the plant possess medicinal properties, but the leaves are a rich source of vitamins A and C, potassium, proteins, calcium, and iron. It is also rich in phytochemicals such as carotenoids, alkaloids, and flavonoids, as well as amino acids such as cysteine, lysine, methionine, and tryptophan [78, 79]. Hence, it serves as an alternative source of nutritional supplement [78, 160].

In Nigeria, where it is called “Zogalla” by the Hausas, “Gbogbonise” and “Okochi egbu” by the Yoruba- and Igbo-speaking tribes, respectively, the whole plant serves as a source of food, medicine, fodder, fencing, firewood, gum and coagulant for water purification. The leaves are medicinally used to treat malaria, typhoid fever, high blood pressure, arthritis, swellings, hypertension, and diabetes, as well as to elicit lactation and boost the immune system. It is also used for the treatment of cancer, either by boiling it alone or combined with lemon grass (*Cymbopogon ciratus*) and bitter leaf (*Vernonia amygdalina*) [161].

The nutritional, antimicrobial [162, 163], antioxidant [164], hypocholesterolemic [165], hepatoprotective [166], anti-diabetic [167, 168], and anticancer [76–78, 83, 85] properties have been reported. Extracts of leaves have been shown to be effective anticancer agents. Jung [76] reported that the aqueous extract of the leaves induced apoptosis, inhibited tumor cell growth, and lowered levels of reactive oxygen species (ROS) in human lung cancer cells. The aqueous extract was found to be cytotoxic against Pac-1 (IC_50_: 1.1 mg/mL), COLO 357 (IC_50_: 1.8 mg/mL), and P34 (IC_50_: 1.5 mg/mL) cell lines [81], and moderately cytotoxic against SW48 (IC_50_: 105.47 ± 23.50 μg/mL) [82]. In another study, Sreelatha et al. [77] demonstrated the antiproliferative and apoptosis-inducing effects of the leaves on the KB cells. Several compounds isolated from the leaves have been linked to the anticancer properties of the plant [160]. Guevara et al. [85] isolated four compounds with potential anticancer activity from the ethanol extract of the leaves, with three showing significant activity: Niazimicin **(87)**, *β*-sitosterol-3-O-*β*-d-glucopyranoside **(88)**, and 4(*α*-l-rhamnosyloxy)-benzyl isothiocyanate **(89)**, with IC_50_ values of 35.3 μg/mL, 27.9 μg/mL and 32.7 μg/mL, respectively. Glucomoringin **(86)**, a common glucosinolate found in *M. oleifera*, has also been shown to be cytotoxic against the H460 wild-type (IC_50_: 29.07 ± 0.76 µΜ) and MCF7 (IC_50_: 21.08 ± 5.67 µΜ) cell lines [84].

Some researchers have attributed the antiproliferative potential of *M. oleifera* to its ability to induce ROS in cancer cells, leading to apoptosis [77]. Others reported its ability to lower internal ROS, inhibit cell growth, and induce apoptosis [76]. These are supported by the upregulation of caspase-3 and caspase-9 of the apoptotic pathway [160]. A study by Tiloke et al. [79] showed that *M. oleifera* exerts its antiproliferative effects by increasing oxidative stress, causing DNA fragmentation and inducing apoptosis.

### *Olax mannii* Oliv. (Olacaceae)

**Figure 9:**
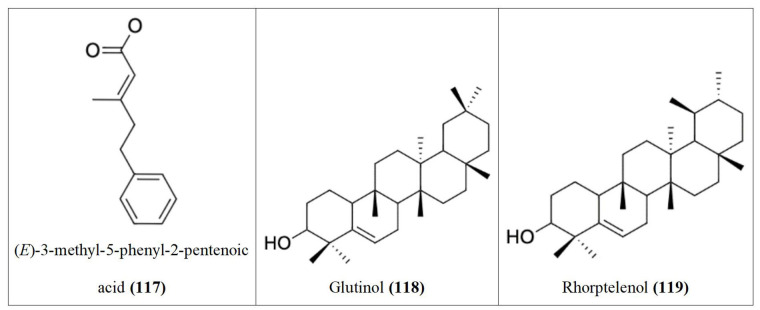
Compounds **117–119** isolated from *Olax mannii* Oliv.

*O. mannii* is a climbing shrub from the Olacaceae family, found mostly in tropical regions, especially in Nigeria, Ghana, and Sierra Leone. The plant is called “Ngborogwu arua” by the Igbos and “Tsada biri” by the Hausas in Eastern and Northern Nigeria, respectively [88, 169]. It is used as an antidote for snakebite and as a treatment for fever [170], cancer, and inflammation [88]. The roots are infused in water or honey and boiled and the infusion is taken for the treatment of depression [171]. Studies have demonstrated the hypoglycemic [170] and antimicrobial activities of the root bark extract of the plant [172]. The leaves, fruits, and root bark have been shown to contain coumarins, steroid/triterpenes, saponins, fatty acids, and tannins; flavonoids and volatile oils are present in the fruits and leaves, while the whole plant is devoid of [173]. Sule et al. [174] Isolated (*E*)-3-methyl-5-phenyl-2-pentenoic acid **(117)** from the petroleum ether extract of the leaves, and two terpenoids, Glutinol **(118)** and Rhorptelenol **(119)** (Figure 9), were isolated from the acetone extract [174]. There are very few reports on the cytotoxic activity of the plant; however, a report by Okoye et al. [88] demonstrated that kaempferol 3-O-*α*-*L*-rhamnopyranoside **(90)**, a flavonoid glycoside isolated from the leaves of *O. manni,* was cytotoxic against human K562 chronic myelogenous leukemia cells, with an IC_50_ value of 50 μM.

### *Parkia biglobosa* (Jacq.) Benth. (Mimosaceae)

*P. biglobosa* (Figure 8B), popularly called “African locust bean tree,” belongs to the family Mimosaceae, and is found in many countries, especially West African countries. It is a perennial, deciduous tree standing between 7 m to 30 m in height, with a large crown and low branches. It has pink-brown to dark-brown pods containing seeds embedded in a yellow pericarp [175]. The seeds make the plant top the list of acceptable, indigenous multipurpose trees in Nigeria and are usually fermented to produce a protein-rich, strong-smelling, tasty condiment popular in many West African countries [176] to improve the taste of soups [177]. In Nigeria, this condiment is called “Iru” by the “Yorubas” [178] and “Daddawa” by the “Hausas” [175]. It is also used for apiculture, fodder, tanning, and in traditional medicine for the treatment of various diseases, including toothaches, diarrhea, bronchitis, cough [175], and arterial hypertension [178]. Studies have demonstrated its antimicrobial, gastroprotective [176], antihypertensive, and antioxidant [177, 178] activities.

It is also used for the treatment of cancer in Southwestern Nigeria [45, 101]. Studies have provided evidence for the cytotoxic effect of the plant [89, 179], with Adetutu et al. [89] providing evidence that the petroleum ether and ethyl acetate fractions of the stem bark of the plant may be a potential source of anticancer drugs, as they were cytotoxic against the SK-LU-1 lung carcinoma cell line, with IC_50_ values of 5.4 ± 0.10 μg/mL and 9.0 ± 0.34 μg/mL, respectively. Also, the methanol extract has been reported to be cytotoxic against PC-3 (IC_50_: 56.1 ± 0.45 μg/mL) cell lines [45].

**Figure 10:**
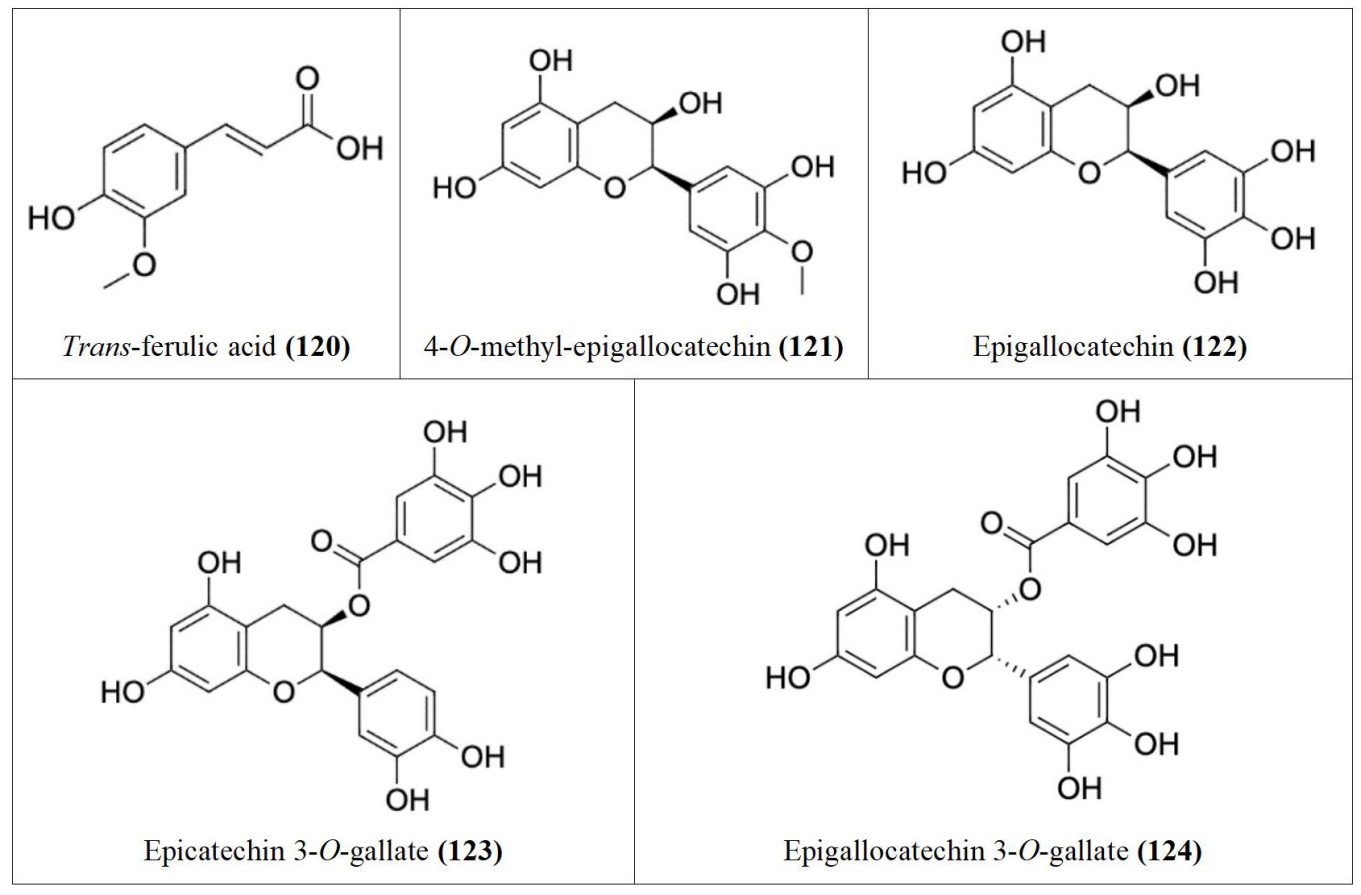
Isolated compounds **120–124** from *Parkia biglobosa*

Like all plants, the therapeutic properties of *P. biglobosa* have been attributed to its constituent phytochemicals such as saponins, tannins, phenolics, and cardiac glycosides [178]. Tala et al. [176] demonstrated that the roots and bark of the plant contain up to forty proanthocyanidins with significant antioxidant activities. Proanthocyanidins are condensed tannins shown to play important roles in disease prevention and have been reported to possess antioxidant, anticancer, anti-ulcerogenic, and anti-inflammatory properties [176]. Other bioactive constituents in the bark are *trans*-ferulic acid **(120)**, a mixture of long-chain cis-ferulates; Lupeol **(58)**, 4-*O*-methyl-epigallocatechin **(121)**, Epigallocatechin **(122)**, Epicatechin 3-*O*-gallate **(123)**, and Epigallocatechin 3-*O*-gallate **(124)** (Figure 10) [180].

### *Peristrophe bicalyculata* (Retz.) Nees (Acanthaceae)

*P. bicalyculata* (Figure 8C), commonly referred to as “Goddess of Mercy,” belongs to the family Acanthaceae [91]. It is native to the Sahel part of Africa, from Mauritania to Niger and Northern Nigeria, India, Burma and Thailand. The Hausas in Northern Nigeria calls it “tubanin dawaki”, meaning “flower of the horse” [90]. In the Indore district of India, the local name is “chotiharjori” [181]. *P. bicalyculata* is used by traditional healers for the treatment of many skin-related ailments, as an antidote for snake poison when macerated in an infusion of rice, and as an insect repellant. It is also used as horse feed and plowed into the soil as green manure. The leaf extract is used for fever, cough and colds, and ear and eye treatments [182]. The leaves of the plant have analgesic, antipyretic, anti-inflammatory, antibacterial, fungistatic, bacteriostatic [183], antihypertensive [184], anticancer [91], and antioxidant properties [185].

A partially purified fraction of the plant inhibited the growth of human oral epidermal carcinoma (KB) cells significantly, with an IC_50_ value of 3.50 ± 0.21 µg/mL, not different from that of cisplatin (3.32 ± 0.09 µg/mL). The fraction induced apoptosis in KB cells after 24 h and 48 h in a concentration-dependent manner [90]. Other studies have demonstrated the anticancer activity of oils from *P. bicalyculata* using MCF-7 (human breast tumor) and MDA-MB-468 (human breast tumor) cells [91], while crude extracts of the plant were cytotoxic against the EAC cell line (EACC) [186, 187].

**Figure 11:**
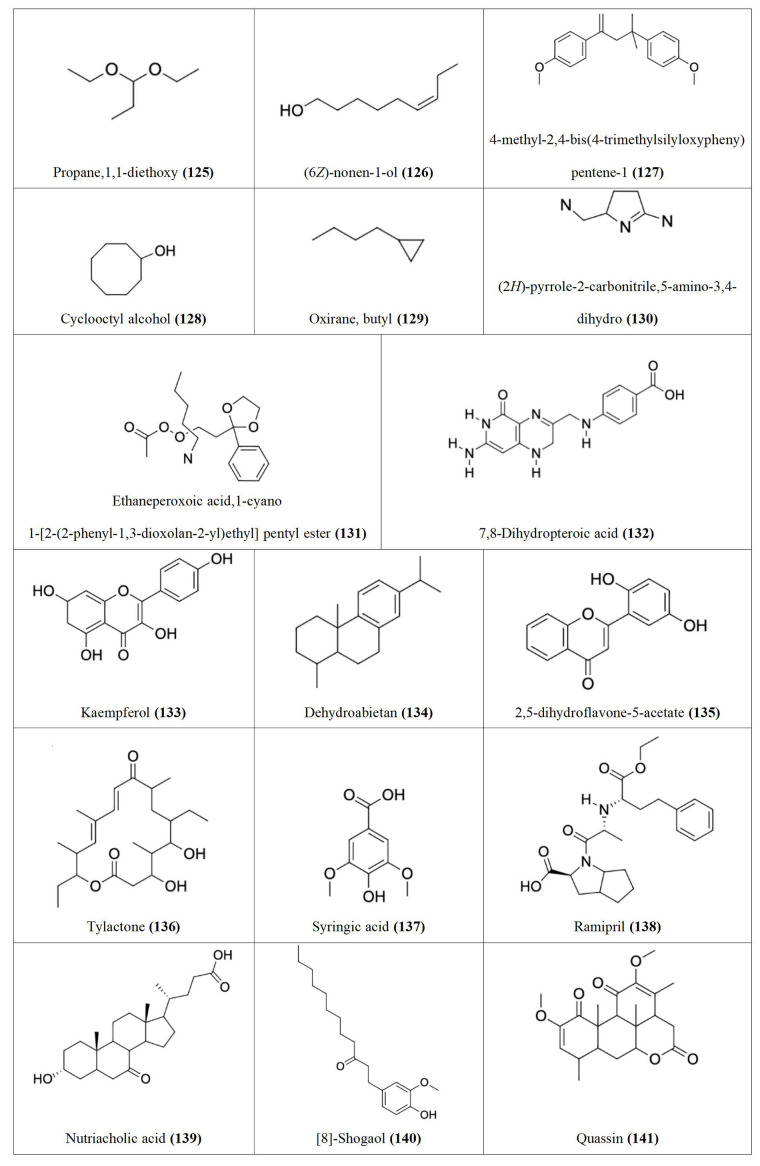
Compounds **125–141** isolated from *Peristrophe bicalyculata*

The GC-MS analysis of the ethanol extract of the plant provided different peaks determining the presence of seven different phytochemicals, namely Propane,1,1-diethoxy **(125)** (68.89%), (6*Z*)-nonen-1-ol **(126)** (24.00%), 4-methyl-2,4-bis(4'-trimethylsilyloxyphenyl)pentene-1 **(127)** (3.56%), Cyclooctyl alcohol **(128)** (1.78%), Oxirane, butyl **(129)** (0.89%), (2*H*)pyrrole-2-carbonitrile,5-amino-3,4-dihydro **(130)** (0.44%) and Ethaneperoxoic acid,1-cyano 1-[2-(2-phenyl-1,3-dioxolan-2-yl)ethyl] pentyl ester **(131)** (0.44%) (Figure 11) [183].

The chloroform, ethyl acetate and methanol extracts of the plant reduced cell viability, with IC_50_ values of 6.21 ± 0.70 µg/mL, 23.39 ± 3.92 µg/mL, and 22.43 ± 3.58 µg/mL, respectively, in HeLa cells; and 1.98 ± 0.33 µg/mL, 8.57 ± 1.91 µg/mL and 28.24 ± 5.57 µg/mL, respectively in MRC5-SV2 cancer cells. Using LC-tandem MS analysis, the following constituents were identified in the chloroform extract: 7,8-Dihydropteroic acid **(132)**, Kaempferol **(133)**, Dehydroabietan **(134)** and 2,5-dihydroflavone-5-acetate **(135)**, while the ethylacetate extract contained Tylactone **(136)**, Syringic acid **(137)**, Ramipril **(138)**, Nutriacholic acid **(139)**, [8]-Shogaol **(140)** and Quassin **(141)** [92] (Figure 11).

### *Vitex doniana* Sw. (Verbenaceae)

Vitex is the largest genus in the family Verbenaceae, comprising 250 species and distributed all over the world. The Vitex species are deciduous shrubs, most of which are used in medicine for the treatment of various diseases [188]. In Nigeria, it is used for gastroenteritis, diarrhea, cancer and as an antimicrobial [45]. Its antioxidant [189] and antiplasmodial [190] activities have also been reported. The fresh leaves contain saponins, tannins, anthraquinones, terpenoids, flavonoids, and alkaloids [189]. An ethnomedicinal approach used by Fadeyi et al. [45] selecting medicinal plants native to Nigeria demonstrated that the methanol extract of the bark and root of *V. doniana* (Figure 8D) inhibited the growth of BT-549, BT-20, PC-3, SW-80 and Jurkat cancer cell lines [45]. The methanol extract of the bark was cytotoxic against BT-549 (IC_50_: 62.5 ± 0.23 μg/mL), BT-20 (IC_50_: 171.1 ± 1.33 μg/mL), SW-80 (IC_50_: 89.2 ± 6.65 μg/mL) and Jurkat (IC_50_: 84.0 ± 1.13 μg/mL) cells, while the IC_50_ values for the root extract were: BT-549 (IC_50_: 44.9 ± 0.10 μg/mL), BT-20 (IC_50_: 152.3 ± 1.22 μg/mL), PC-3 (IC_50_: 177.3 ± 1.01 μg/mL), SW-80 (IC_50_: 45.6 ± 1.35 μg/mL) and Jurkat (IC_50_: 43.4 ± 0.64 μg/mL) [45].

## Conclusions

This review identified forty-six plants used in the treatment of cancer in various parts of Nigeria and documents data on the potential anticancer effects of these plants. The cytotoxic activities of extracts from the plants have been determined, but very few have been fully investigated to identify active anticancer constituents. Compounds with significant cytotoxic activities have been isolated from *Acanthospermum hispidum*, *Ageratum conyzoides*, *Annona muricata*, *C. aurantium, C. anisata*, *E. suaveolens*, *K. pinnata*, *F. zanthoxyloides*, *Lecaniodiscus cupanioides*, *M. oleifera* and *Olax mannii.* However, the mechanisms of action of some isolated constituents have not been established. It is important that research on anticancer activities of plants does not stop at identifying the active constituents, but advances to the development of leads and clinical trials of the resulting drug candidates. The investigated medicinal plants presented in this article could serve as a starting point. Pre-clinical investigations should also consider potential synergies between the compounds, as well as the development of herbal recipes with highly defined compositions for the potential treatment of cancers.

## Abbreviations

EAC: Ehrlich ascites carcinoma
GC-MS: gas chromatography-mass spectrometry
RD: rhabdomyosarcoma
ROS: reactive oxygen species
WHO: World Health Organization

## Declarations

### Availability of data and materials

The primary data for this review were sourced online from databases listed in the article. Referenced articles are accessible on PubMed, Science Direct, Scopus, Google Scholar, and Web of Science. Additional supporting data are available from the corresponding author upon request.

### Copyright

© The Author(s) 2024.

## References

[1] Vogelstein B, Kinzler KW (2004). Cancer genes and the pathways they control. Nat Med.

[2] Jemal A, Siegel R, Xu J, Ward E (2010). Cancer statistics, 2010. CA Cancer J Clin.

[3] Sultana S, Asif HM, Nazar HMI, Akhtar N, Rehman JU, Rehman RU (2014). Medicinal plants combating against cancer--a green anticancer approach. Asian Pac J Cancer Prev.

[4] Abdullahi AD, Mustapha RK, Yau S, Adam MS (2018). Exploring the Nigerian Medicinal Plants with Anticancer Activities: A Pharmacological Review. Modern Chemistry.

[5] Sung H, Ferlay J, Siegel RL, Laversanne M, Soerjomataram I, Jemal A (2021). Global Cancer Statistics 2020: GLOBOCAN Estimates of Incidence and Mortality Worldwide for 36 Cancers in 185 Countries. CA Cancer J Clin.

[6] WHO report on the global tobacco epidemic, 2008: the MPOWER package [Internet]. https://www.who.int/publications/i/item/9789241596282.

[7] World Cancer Report 2014 [Internet]. https://publications.iarc.fr/Non-Series-Publications/World-Cancer-Reports/World-Cancer-Report-2014.

[8] (2016). Osaro, E.

[9] Ishola F, Omole O (2016). A vision for improved cancer screening in Nigeria. Lancet Glob Health.

[10] Cancer control: a global snapshot in 2015 [Internet]. https://www.who.int/publications/i/item/cancer-control-a-global-snapshot-in-2015.

[11] Global Status Report on Alcohol and Health 2014 [Internet]. https://www.who.int/publications/i/item/global-status-report-on-alcohol-and-health-2014.

[12] Huang M, Lu J, Ding J (2021). Natural Products in Cancer Therapy: Past, Present and Future. Nat Prod Bioprospect.

[13] Mazumder K, Aktar A, Roy P, Biswas B, Hossain ME, Sarkar KK (2022). A Review on Mechanistic Insight of Plant Derived Anticancer Bioactive Phytocompounds and Their Structure Activity Relationship. Molecules.

[14] Ohiagu FO, Chikezie PC, Chikezie CM, Enyoh CE (2021). Anticancer activity of Nigerian medicinal plants: a review. Futur J Pharm Sci.

[15] (2001). Rocha ABd, Lopes RM, Schwartsmann G. Natural products in anticancer therapy. Curr Opin Pharmacol.

[16] Newman DJ, Cragg GM (2016). Natural Products as Sources of New Drugs from 1981 to 2014. J Nat Prod.

[17] Li C, Huang S, Wu M, Chen Y, Tsang S, Chyuan J (2012). Induction of apoptosis by ethanolic extract of Corchorus olitorius leaf in human hepatocellular carcinoma (HepG2) cells via a mitochondria-dependent pathway. Molecules.

[18] Taiwo BJ, Taiwo GO, Olubiyi OO, Fatokun AA (2016). Polyphenolic compounds with anti-tumour potential from Corchorus olitorius (L.) Tiliaceae, a Nigerian leaf vegetable. Bioorg Med Chem Lett.

[19] Weir M What is complementary and alternative medicine.

[20] Snyder L Carroll, RJ. History, Definitions, and What Is It Today?. Complementry and Alternative Medicine.

[21] Eisenberg DM, Kessler RC, Rompay MIV, Kaptchuk TJ, Wilkey SA, Appel S (2001). Perceptions about complementary therapies relative to conventional therapies among adults who use both: results from a national survey. Ann Intern Med.

[22] Bussmann RW, Glenn A (2011). Medicinal plants used in Northern Peru for the treatment of bacterial and fungal infections and inflammation symptoms. J Med Plants Res.

[23] Ogbourne SM, Parsons PG (2014). The value of nature's natural product library for the discovery of New Chemical Entities: the discovery of ingenol mebutate. Fitoterapia.

[24] Wannes WA, Tounsi MS, Marzouk B (2017). A review of Tunisian medicinal plants with anticancer activity. J Complement Integr Med.

[25] Amri E (2014). The role of selected plant families with dietary Ethnomedicinal species used as anticancer. J Med Plants.

[26] Fouche G, Cragg GM, Pillay P, Kolesnikova N, Maharaj VJ, Senabe J (2008). *In vitro* anticancer screening of South African plants. J Ethnopharmacol.

[27] Zschocke S, Rabe T, Taylor JL, Jäger AK, Staden Jv (2000). Plant part substitution – a way to conserve endangered medicinal plants?. J Ethnopharmacol.

[28] Vardiman JW, Thiele J, Arber DA, Brunning RD, Borowitz MJ, Porwit A (2009). The 2008 revision of the World Health Organization (WHO) classification of myeloid neoplasms and acute leukemia: rationale and important changes. Blood.

[29] (2007). World Cancer Research Fund / American Institute for Cancer Research.

[30] Akindele AJ, Wani Z, Mahajan G, Sharma S, Aigbe FR, Satti N (2014). Anticancer activity of *Aristolochia ringens* Vahl. (Aristolochiaceae). J Tradit Complement Med.

[31] Tiwary BK, Bihani S, Kumar A, Chakraborty R, Ghosh R (2015). The *in vitro* cytotoxic activity of ethno-pharmacological important plants of Darjeeling district of West Bengal against different human cancer cell lines. BMC Complement Altern Med.

[32] Kuete V, Efferth T (2015). African flora has the potential to fight multidrug resistance of cancer. Biomed Res Int.

[33] Solowey E, Lichtenstein M, Sallon S, Paavilainen H, Solowey E, Lorberboum-Galski H (2014). Evaluating medicinal plants for anticancer activity. ScientificWorldJournal.

[34] Collaboration GBoDC, Fitzmaurice C, Allen C, Barber RM, Barregard L, Bhutta ZA (2017). Global, Regional, and National Cancer Incidence, Mortality, Years of Life Lost, Years Lived With Disability, and Disability-Adjusted Life-years for 32 Cancer Groups, 1990 to 2015: A Systematic Analysis for the Global Burden of Disease Study. JAMA Oncol.

[35] Sofowora A (1993). Recent trends in research into African medicinal plants. J Ethnopharmacol.

[36] Gbile ZO, Adesina SK (1987). Nigerian flora and its pharmaceutical potential. J Ethnopharmacol.

[37] Adelaja A (2006). Nigeria boosts research into traditional medicine. SciDev. net-Enterprise.

[38] Ashidi JS, Houghton PJ, Hylands PJ, Efferth T (2010). Ethnobotanical survey and cytotoxicity testing of plants of South-western Nigeria used to treat cancer, with isolation of cytotoxic constituents from *Cajanus cajan* Millsp. leaves. J Ethnopharmacol.

[39] Ogbole OO, Segun PA, Adeniji AJ (2017). In vitro cytotoxic activity of medicinal plants from Nigeria ethnomedicine on Rhabdomyosarcoma cancer cell line and HPLC analysis of active extracts. BMC Complement Altern Med.

[40] Mothana RA, Lindequist U, Gruenert R, Bednarski PJ (2009). Studies of the in vitro anticancer, antimicrobial and antioxidant potentials of selected Yemeni medicinal plants from the island Soqotra. BMC Complement Altern Med.

[41] Oni MO, Bello OO, Ademola RA, Mba UD, Oni VO (2022). Anticancer and antibacterial potentials of methanolic extracts of the leaf and stem bark of *Afzelia Africana*. Egypt Pharm J.

[42] Daffalha AAE, Mona A A (2015). Antioxidant activity and Invitro Cyto-toxicity of different extracts of Afzelia africana bark. Am J Res Commun.

[43] Adebayo AH, Tan NH, Akindahunsi AA, Zeng GZ, Zhang YM (2010). Anticancer and antiradical scavenging activity of Ageratum conyzoides L. (Asteraceae). Pharmacogn Mag.

[44] Adebayo AH, Jig C, Zhang Y, He W, Zeng G, Han H (2011). A new chromene isolated from Ageratum conyzoides. Nat Prod Commun.

[45] Fadeyi SA, Fadeyi OO, Adejumo AA, Okoro C, Myles EL (2013). *In vitro* anticancer screening of 24 locally used Nigerian medicinal plants. BMC Complement Altern Med.

[46] Taiwo BJ, Fatokun AA, Olubiyi OO, Bamigboye-Taiwo OT, Heerden FRv, Wright CW (2017). Identification of compounds with cytotoxic activity from the leaf of the Nigerian medicinal plant, *Anacardium occidentale* L. (Anacardiaceae). Bioorg Med Chem.

[47] Yang C, Gundala SR, Mukkavilli R, Vangala S, Reid MD, Aneja R (2015). Synergistic interactions among flavonoids and acetogenins in Graviola (*Annona muricata*) leaves confer protection against prostate cancer. Carcinogenesis.

[48] Gavamukulya Y, Abou-Elella F, Wamunyokoli F, AEl-Shemy H (2014). Phytochemical screening, anti-oxidant activity and *in vitro* anticancer potential of ethanolic and water leaves extracts of *Annona muricata* (Graviola). Asian Pac J Trop Med.

[49] Najmuddin SUFS, Romli MF, Hamid M, Alitheen NB, Rahman NMANA (2016). Anti-cancer effect of Annona Muricata Linn Leaves Crude Extract (AMCE) on breast cancer cell line. BMC Complement Altern Med.

[50] Moghadamtousi SZ, Kadir HA, Paydar M, Rouhollahi E, Karimian H (2014). *Annona muricata* leaves induced apoptosis in A549 cells through mitochondrial-mediated pathway and involvement of NF-κB. BMC Complement Altern Med.

[51] Moghadamtousi SZ, Karimian H, Rouhollahi E, Paydar M, Fadaeinasab M, Kadir HA (2014). *Annona muricata* leaves induce G₁ cell cycle arrest and apoptosis through mitochondria-mediated pathway in human HCT-116 and HT-29 colon cancer cells. J Ethnopharmacol.

[52] Pieme CA, Kumar SG, Dongmo MS, Moukette BM, Boyoum FF, Ngogang JY (2014). Antiproliferative activity and induction of apoptosis by *Annona muricata* (Annonaceae) extract on human cancer cells. BMC Complement Altern Med.

[53] Umeokoli BO, Onyegbule FA, Okoye FBC, Wang H, Kalscheuer R, Müller WEG (2017). New amide and dioxopiperazine derivatives from leaves of *Breynia nivosa*. Fitoterapia.

[54] El-Manawaty M, Fayad W, El-Fiky NM, Wassel GM, El-Menshawi BS (2013). High-throughput screening of 75 euphorbiaceae and myrtaceae plant extracts for in-vitro antitumor and pro-apoptotic activities on human tumor cell lines, and lethality to brine shrimp. Int J Pharm Pharm Sci.

[55] Erharuyi O, Adhikari A, Falodun A, Jabeen A, Imad R, Ammad M (2017). Cytotoxic, Anti-inflammatory, and Leishmanicidal Activities of Diterpenes Isolated from the Roots of *Caesalpinia pulcherrima*. Planta Med.

[56] Adaramoye O, Erguen B, Oyebode O, Nitzsche B, Höpfner M, Jung K (2015). Antioxidant, antiangiogenic and antiproliferative activities of root methanol extract of *Calliandra portoricensis* in human prostate cancer cells. J Integr Med.

[57] Oyebode OT, Owumi SE, Oyelere AK, Olorunsogo OO (2019). *Calliandra portoricensis* Benth exhibits anticancer effects via alteration of Bax/Bcl-2 ratio and growth arrest in prostate LNCaP cells. J Ethnopharmacol.

[58] Lee D, Park K, Park H, Kang S, Nagappan A, Kim J (2012). Flavonoids Isolated from Korea *Citrus aurantium* L. Induce G2/M Phase Arrest and Apoptosis in Human Gastric Cancer AGS Cells. Evid Based Complement Alternat Med.

[59] Park KI, Park HS, Nagappan A, Hong GE, Lee DH, Kang SR (2012). Induction of the cell cycle arrest and apoptosis by flavonoids isolated from Korean *Citrus aurantium* L. in non-small-cell lung cancer cells. Food Chem.

[60] Lee SH, Yumnam S, Hong GE, Raha S, Saralamma VVG, Lee HJ (2015). Flavonoids of Korean *Citrus aurantium* L. Induce Apoptosis via Intrinsic Pathway in Human Hepatoblastoma HepG2 Cells. Phytother Res.

[61] Tatsimo SJN, Lamshöft M, Mouafo FT, Lannang AM, Sarkar P, Bag PK (2015). LC-MS guided isolation of antibacterial and cytotoxic constituents from *Clausena anisata*. Medicinal Chemistry Research.

[62] Ikpefan EO, Ayinde BA, Omeje EO, Azhar M, Farooq AD, Shah ZA (2021). Isolation and anti-cancer evaluation of two anti-proliferative constituents from the chloroform fraction of leaves of *Conyza Sumatrensis* (Retz.) E. H. Walker, Asteraceae. Sci Afr.

[63] Hansakul P, Ngamkitidechakul C, Ingkaninan K, Sireeratawong S, Panunto W (2009). Apoptotic induction activity of Dactyloctenium aegyptium (L.) P.B. and Eleusine indica (L.) Gaerth. extracts on human lung and cervical cancer cell lines. Songklanakarin J Sci Technol.

[64] Sowemimo A, Venables L, Odedeji M, Koekemoer T, Venter Mvd, Hongbing L (2015). Antiproliferative mechanism of the methanolic extract of *Enterolobium cyclocarpum* (Jacq.) Griseb. (Fabaceae). J Ethnopharmacol.

[65] Comoë L, Jeannesson P, Trentesaux C, Desoize B, Jardillier JC (1987). The antileukemic alkaloid fagaronine and the human K 562 leukemic cells: effects on growth and induction of erythroid differentiation. Leuk Res.

[66] Kassim OO, Copeland RL, Kenguele HM, Nekhai S, Ako-Nai KA, Kanaan YM (2015). Antiproliferative activities of Fagara xanthoxyloides and Pseudocedrela kotschyi against prostate cancer cell lines. Anticancer Res.

[67] Osamudiamen PM, Aiyelaagbe OO, Vaid S, Saxena AK (2018). Comparative Evaluation of the Anti-cancer Activities of the Crude Extracts of Four Nigerian Chewing Sticks. J Biol Act Prod Nat.

[68] Adedokun O, Ntungwe EN, Viegas C, Ayinde BA, Barboni L, Maggi F (2022). Enhanced Anticancer Activity of *Hymenocardia acida* Stem Bark Extract Loaded into PLGA Nanoparticles. Pharmaceuticals (Basel).

[69] Fadayomi IE, Johnson-Ajinwo OR, Pires E, McCullagh J, Claridge TDW, Forsyth NR (2021). Clerodane Diterpenoids from an Edible Plant *Justicia insularis*: Discovery, Cytotoxicity, and Apoptosis Induction in Human Ovarian Cancer Cells. Molecules.

[70] Atolani O, Olatunji GA, Fabiyi OA, Adeniji AJ, Ogbole OO (2013). Phytochemicals from *Kigelia pinnata* leaves show antioxidant and anticancer potential on human cancer cell line. J Med Food.

[71] Jackson SJ, Houghton PJ, Retsas S, Photiou A (2000). *In vitro* cytotoxicity of norviburtinal and isopinnatal from *Kigelia pinnata* against cancer cell lines. Planta Med.

[72] Adesegun SA, Coker HA, Hamann MT (2014). Anti-cancerous triterpenoid saponins from *Lecaniodiscus cupanioides*. J Nat Prod (Gorakhpur).

[73] Das K, Yasin M, Mahbub NU, Islam MS, Mahbuba N (2014). Evaluation of antioxidant and cytotoxic activity of methanolic extract of* Mimosa pudica* leaves. The Pharma Innovat.

[74] Chowdhury S, Saha D, Paul S (2012). IN VITRO CYTOTOXIC ACTIVITIES OF METHANOLIC EXTRACT OF MIMOSA PUDICA. Bull Pharm Res.

[75] Jose J, Dhanya AT, Haridas KR, Kumar TMS, Jayaraman S, Variyar EJ (2016). Structural characterization of a novel derivative of myricetin from *Mimosa pudica* as an anti-proliferative agent for the treatment of cancer. Biomed Pharmacother.

[76] Jung IL (2014). Soluble extract from *Moringa oleifera* leaves with a new anticancer activity. PLoS One.

[77] Sreelatha S, Jeyachitra A, Padma PR (2011). Antiproliferation and induction of apoptosis by Moringa oleifera leaf extract on human cancer cells. Food Chem Toxicol.

[78] Charoensin S (2014). Antioxidant and anticancer activities of Moringa oleifera leaves. J Med Plants Res.

[79] Tiloke C, Phulukdaree A, Chuturgoon AA (2013). The antiproliferative effect of *Moringa oleifera* crude aqueous leaf extract on cancerous human alveolar epithelial cells. BMC Complement Altern Med.

[80] Krishnamurthy PT, Vardarajalu A, Wadhwani A, Patel V (2015). Identification and characterization of a potent anticancer fraction from the leaf extracts of Moringa oleifera L. Indian J Exp Biol.

[81] Berkovich L, Earon G, Ron I, Rimmon A, Vexler A, Lev-Ari S (2013). *Moringa Oleifera* aqueous leaf extract down-regulates nuclear factor-kappaB and increases cytotoxic effect of chemotherapy in pancreatic cancer cells. BMC Complement Altern Med.

[82] Pamok S, Vinitketkumnuen SSU, Saenphet K (2012). Antiproliferative effect of Moringa oleifera Lam. and Pseuderanthemum palatiferum (Nees) Radlk extracts on the colon cancer cells. J Med Plants Res.

[83] Elsayed EA, Sharaf-Eldin MA, Wadaan M (2015). In vitro Evaluation of Cytotoxic Activities of Essential Oil from Moringa oleifera Seeds on HeLa, HepG2, MCF-7, CACO-2 and L929 Cell Lines. Asian Pac J Cancer Prev.

[84] Brunelli D, Tavecchio M, Falcioni C, Frapolli R, Erba E, Iori R (2010). The isothiocyanate produced from glucomoringin inhibits NF-kB and reduces myeloma growth in nude mice *in vivo*. Biochem Pharmacol.

[85] Guevara AP, Vargas C, Sakurai H, Fujiwara Y, Hashimoto K, Maoka T (1999). An antitumor promoter from *Moringa oleifera* Lam. Mutat Res.

[86] Bose CK (2007). Possible role of Moringa oleifera Lam. root in epithelial ovarian cancer. MedGenMed.

[87] Charles-Okhe O, Odeniyi MA, Fakeye TO, Ogbole OO, Akinleye TE, Adeniji AJ (2022). Cytotoxic activity of crude extracts and fractions of African peach (nauclea latifolia smith) stem bark on two cancer cell lines. Phytomedicine Plus.

[88] Okoye FBC, Sawadogo WR, Sendker J, Aly AH, Quandt B, Wray V (2015). Flavonoid glycosides from *Olax mannii*: Structure elucidation and effect on the nuclear factor kappa B pathway. J Ethnopharmacol.

[89] Adetutu A, Morgan WA, Corcoran O, Chimezie F (2012). RETRACTED: Antibacterial activity and in vitro cytotoxicity of extracts and fractions of Parkia biglobosa (Jacq.) Benth. stem bark and Ageratum conyzoides Linn. leaves. Environ Toxicol Pharmacol.

[90] Abdulazeez M, Ibrahim S, Ameh D, Ayo J, Carvalho L (2013). ANTICANCER ACTIVITIES OF EXTRACTS OF Peristrophe bicalyculata (RETZ) NEES. Rom Biotechnol Lett.

[91] Ogunwande IA, Walker TM, Bansal A, Setzer WN, Essien EE (2010). Essential oil constituents and biological activities of Peristrophe bicalyculata and Borreria verticillata. Nat Prod Commun.

[92] Abdulazeez MA, Jasim HA, Bashir M, Ross K, Fatokun AA (2022). *Peristrophe bicalyculata* (Retz) Nees contains principles that are cytotoxic to cancer cells and induce caspase-mediated, intrinsic apoptotic death through oxidative stress, mitochondrial depolarisation and DNA damage. Biomed Pharmacother.

[93] Essien EE, Ogunwande IA, Setzer WN, Ekundayo O (2012). Chemical composition, antimicrobial, and cytotoxicity studies on *S. erianthum* and *S. macranthum* essential oils. Pharm Biol.

[94] Oladimeji AO, Aliyu MB, Ogundajo AL, Babatunde O, Adeniran OI, Balogun OS (2016). Identification and comparison of the volatile constituents of fresh and dried leaves of *Spondias mombin* found in North-central Nigeria:* in vitro* evaluation of their cytotoxic and antioxidant activities. Pharm Biol.

[95] Oyeyemi IT (2013). Bakare AA. Genotoxic and anti-genotoxic effect of aqueous extracts of *Spondias mombin* L., *Nymphea lotus* L. and *Luffa cylindrica* L. on* Allium cepa* root tip cells. Caryologia.

[96] Idu M, Onyibe H (2007). Medicinal plants of Edo state, Nigeria. RJMP.

[97] Uche FI, Drijfhout FP, McCullagh J, Richardson A, Li W (2016). Cytotoxicity Effects and Apoptosis Induction by Bisbenzylisoquinoline Alkaloids from *Triclisia subcordata*. Phytother Res.

[98] Wong FC, Woo CC, Hsu A, Tan BKH (2013). The anti-cancer activities of *Vernonia amygdalina* extract in human breast cancer cell lines are mediated through caspase-dependent and p53-independent pathways. PLoS One.

[99] Izevbigie EB (2003). Discovery of water-soluble anticancer agents (edotides) from a vegetable found in Benin City, Nigeria. Exp Biol Med (Maywood).

[100] Taylor P, Colman L, Bajoon J (2014). The search for plants with anticancer activity: pitfalls at the early stages. J Ethnopharmacol.

[101] Segun PA, Ogbole OO, Ajaiyeoba EO (2018). Medicinal plants used in the management of cancer among the Ijebus of southwestern Nigeria. J Herb Med.

[102] Sebaugh JL (2011). Guidelines for accurate EC50/IC50 estimation. Pharm Stat.

[103] Rixe O, Fojo T (2007). Is cell death a critical end point for anticancer therapies or is cytostasis sufficient?. Clin Cancer Res.

[104] Chakraborty AK, Gaikwad AV, Singh KB (2012). Phytopharmacological review on *Acanthospermum hispidum*. JAPS.

[105] Cartagena E, Bardón A, Catalán CA, Hernández ZNd, Hernández LR, Joseph-Nathan P (2000). Germacranolides and a new type of guaianolide from *Acanthospermum hispidum*. J Nat Prod.

[106] Kashina BD, Mabagala RB, Mpunami A (2003). First report of *Ageratum conyzoides* L. and *Sida acuta* Burm F. as new weed hosts of tomato* yellow leaf curl Tanzania virus*. Plant Protect Sci.

[107] Singh HP, Batish DR, Shalinder K, Kohli RK, Dogra KS (2004). Allelopathic interference of Ageratum conyzoides L. against some crop plants.14th Aust. Weeds Conf.

[108] Chahal R, Nanda A, Akkol EK, Sobarzo-Sánchez E, Arya A, Kaushik D (2021). *Ageratum conyzoides* L. and Its Secondary Metabolites in the Management of Different Fungal Pathogens. Molecules.

[109] Moghadamtousi SZ, Fadaeinasab M, Nikzad S, Mohan G, Ali HM, Kadir HA (2015). *Annona muricata* (Annonaceae): A Review of Its Traditional Uses, Isolated Acetogenins and Biological Activities. Int J Mol Sci.

[110] Roslida A, Tay C, Zuraini A, Chan P (2010). Anti-inflammatory and anti-nociceptive activities of the ethanolic extract of *Annona muricata* leaf. Journal of Natural Remedies.

[111] Gajalakshmi S, Vijayalakshmi S, Devi Rajeswari V (2012). PHYTOCHEMICAL AND PHARMACOLOGICAL PROPERTIES OF ANNONA MURICATA: A REVIEW. Int J Pharm Pharm Sci.

[112] Coria-Tellez AV, Montalvo-Gónzalez E, Yahia EM, Obledo-Vázquez EN (2018). *Annona muricata*: A comprehensive review on its traditional medicinal uses, phytochemicals, pharmacological activities, mechanisms of action and toxicity. Arabian J Chem.

[113] Gbadamosi I (2014). The mineral, proximate and phytochemical components of ten Nigerian medicinal plants used in the management of arthritis. Afr J Pharm Pharmacol.

[114] (2016). El-Ghani, MMA. 2016. Traditional medicinal plants of Nigeria: An overview. Agric Biol J N Am.

[115] Ogugu S, Kehinde A, James B, Paul D (2012). Assessment of cytotoxic effects of methanol extract of Calliandra portoricensis using brine shrimp (Artemia salina) lethality bioassay. Glob J Biosciences Biotechnol.

[116] Kosemani SO, Bakare AA, Adaramoye OA (2022). Fraction from *Calliandra portoricensis* reduces 7, 12 dimethylbenz(a)anthracene-induced mammary tumors in Wistar rats. Avicenna J Phytomed.

[117] Barreca D, Bellocco E, Caristi C, Leuzzi U, Gattuso G (2011). Distribution of C- and O-glycosyl flavonoids, (3-hydroxy-3-methylglutaryl)glycosyl flavanones and furocoumarins in *Citrus aurantium* L. juice. Food Chemistry.

[118] Karimi E, Oskoueian E, Hendra R, Oskoueian A, Jaafar HZE (2012). Phenolic compounds characterization and biological activities of *Citrus aurantium* bloom. Molecules.

[119] Segun PA, Ismail FMD, Ogbole OO, Nahar L, Evans AR, Ajaiyeoba EO (2018). Acridone alkaloids from the stem bark of *Citrus aurantium* display selective cytotoxicity against breast, liver, lung and prostate human carcinoma cells. J Ethnopharmacol.

[120] Arbab IA, Abdul AB, Aspollah M, Abdelwahab SI, Ibrahim MY, Ali Z (2012). A review of traditional uses, phytochemical and pharmacological aspects of selected members of *Clausena* genus (Rutaceae). J Med Plants Res.

[121] Gbadamosi IT, Erinoso SM (2016). A review of twenty ethnobotanicals used in the management of breast cancer in Abeokuta, Ogun State, Nigeria. Afr J Pharm Pharmacol.

[122] Aiyegoro OA, Akinpelu DA, Okoh AI (2007). *In vitro* antibacterial potentials of the stem bark of red water tree (*Erythrophleum suaveolens*). J Biol Sci.

[123] Hassan SW, Ladan MJ, Dogondaji RA, Umar RA, Bilbis LS, Hassan LG (2007). Phytochemical and toxicological studies of aqueous leaves extracts of *Erythrophleum africanum*. Pak J Biol Sci.

[124] Dongmo AB, Kamanyi A, Anchang MS, Nkeh BC, Njamen D, Nguelefack TB (2001). Anti-inflammatory and analgesic properties of the stem bark extracts of *Erythrophleum suaveolens* (Caesalpiniaceae), Guillemin & Perrottet. J Ethnopharmacol.

[125] Grkovic T, Evans JR, Akee RK, Guo L, Davis M, Jato J (2019). Erythrofordins D and E, two new cassaine-type diterpenes from *Erythrophleum suaveolens*. Bioorg Med Chem Lett.

[126] Saha JBT, Abia D, Dumarçay S, Ndikontar MK, Gérardin P, Noah JN (2013). Antioxidant activities, total phenolic contents and chemical compositions of extracts from four Cameroonian woods: Padouk (*Pterocarpus soyauxii Taubb*), tali (*Erythrophleum suaveolens*), moabi (*Baillonella toxisperma*), and movingui (*Distemonanthus benthamianus*). Ind Crops Prod.

[127] Saini S, Kaur H, Verma B, Singh SK (2009). Kigelia africana (Lam.) Benth. — An overview. Natural Product Radiance.

[128] Bello I, Shehu MW, Musa M, Asmawi MZ, Mahmud R (2016). *Kigelia africana* (Lam.) Benth. (Sausage tree): Phytochemistry and pharmacological review of a quintessential African traditional medicinal plant. J Ethnopharmacol.

[129] Gabriel OA, Olubunmi A (2009). Comprehensive scientific demystification of *Kigelia africana*: A review. Afr J Pure Appl Chem.

[130] Houghton P, Jâger A (2002). The sausage tree (*Kigelia pinnata*): Ethnobotany and recent scientific work. S Afr J Bo.

[131] Misra LN, Wouatsa NAV, Kumar S, Kumar RV, Tchoumbougnang F (2013). Antibacterial, cytotoxic activities and chemical composition of fruits of two Cameroonian Zanthoxylum species. J Ethnopharmacol.

[132] Adesina S (2005). The Nigerian *Zanthoxylum*: chemical and biological values. Afr J Trad Comp Alt Med.

[133] Couillerot E, Caron C, Trentesaux C, Chenieux J, Audran J, Bajaj YPS (1999). *Fagara zanthoxyloides* Lam. (Rutaceae): In Vitro Culture and the Production of Benzophenanthridine and Furoquinoline Alkaloids. Medicinal and Aromatic Plants XI.

[134] Adefisoye MA, Ajibadeako-Nai K, Bisi-Johnson MA (2012). Phytochemical and antibacterial activity of the extracts of *Fagara zanthoxyloides* on selected cariogenic and enteric bacterial isolates. J Intercult Ethnopharmacol.

[135] Grant WB, Boucher BJ (2011). Low vitamin D status likely contributes to the link between periodontal disease and breast cancer. Breast Cancer Res Treat.

[136] Tin-Wa M, Bell CL, Bevelle C, Fong HH, Farnsworth NR (1974). Potential Anticancer Agents I: Confirming Evidence for the Structure of Fagaronine. J Pharm Sci.

[137] Tillequin F (2007). Rutaceous alkaloids as models for the design of novel antitumor drugs. Phytochem Rev.

[138] Nafiu MO, Abdulsalam TA, Akanji MA (2013). Phytochemical Analysis and Antimalarial Activity Aqueous Extract of *Lecaniodiscus cupanioides* Root. J Trop Med.

[139] Oselebe HO, Nnamani CV, Okporie EO (2013). Ethnobotanical survey of underutilized crops and spices of some local communities in Nigeria: potentials for improved nutrition, food security and poverty reduction. IOSR Journal of Pharmacy.

[140] Ogunmefun OT, Gbile ZO (2012). An ethnobotanical study of anti-rheumatic plants in South Western States of Nigeria. Asian Journal of Science and Technology.

[141] Adeyemi O, Yemitan O, Adeogun O (2024). Analgesic activity of the aqueous root extract *Lecaniodiscus cupanioides*. W Afr J Pharmacol Drug Res.

[142] Yemitan OK, Adeyemi OO (2005). CNS depressant activity of *Lecaniodiscus cupanioides*. Fitoterapia.

[143] Alayande KA, Ashafa AOT (2017). Evaluation of cytotoxic effects and antimicrobial activities of L*ecaniodiscus cupanioides* (Planch.) leaf extract. Tran R Soc S Afr.

[144] Magadula JJ (2014). Phytochemistry and pharmacology of the genus *Macaranga*: a review. J Med Plants Res.

[145] Adesegun S, Elechi N, Coker H (2007). Antioxidant power of *Macaranga barteri* leaf. Am J Food Technol.

[146] Ogundajo AL, Tom Ashafa AO (2019). Chemical profiling, antioxidant and carbohydrate-metabolising enzymes inhibitory potential of fractions from the leaves of *Macaranga bateri* Mull-Arg. Trans R Soc S Afr.

[147] Uduak AE, Kola KA (2010). Antimicrobial activities of some Euphorbiaceae plants used in the traditional medicine of Akwa Ibom State of Nigeria. Ethnobotanical Leaflets.

[148] Ngoumfo RM, Ngounou GE, Tchamadeu CV, Qadir MI, Mbazoa CD, Begum A (2008). Inhibitory effect of macabarterin, a polyoxygenated ellagitannin from *Macaranga barteri*, on human neutrophil respiratory burst activity. J Nat Prod.

[149] Muhammad G, Hussain MA, Jantan I, Bukhari SNA (2016). *Mimosa pudica* L., a High-Value Medicinal Plant as a Source of Bioactives for Pharmaceuticals. Compr Rev Food Sci Food Saf.

[150] Gunawardhana CB, Ranasinghe SJ, Waisundara VY (2015). Review: *Mimosa pudica* Linn.: the garden weed with therapeutic properties. Isr J Plant Sc.

[151] Arokiyaraj S, Sripriya N, Bhagya R, Radhika B, Prameela L, Udayaprakash N (2012). Phytochemical screening, antibacterial and free radical scavenging effects of *Artemisia nilagirica*, *Mimosa pudica* and* Clerodendrum siphonanthus* – An *in*–*vitro* study. Asian Pac J Trop Biomed.

[152] Manoharan S, Kaur J (2013). Anticancer, antiviral, antidiabetic, antifungal and phytochemical constituents of medicinal plants. Am J PharmTech Res.

[153] Sajid I, Bijan K, Zamiul R, Mominul I, Ekramul H (2013). CNS Depressant and Antinociceptive Activities of the Aerial Parts of* Mimosa pudica*. Europ J Appl Sci.

[154] Sowmya A, Ananthi T (2011). Hypolipidemic activity of Mimosa pudica Linn on butter induced hyperlipidemia in rats. Asian J Res Pharm Sci.

[155] Rajendran R, Hemalatha S, Akasakalai K, Madhukrishna CH, Sohil V, Sundaram R (2009). Hepatoprotective activity of Mimosa pudica leaves against Carbontetrachloride induced toxicity. J Nat Prod.

[156] Kokane DD, More RY, Kale MB, Nehete MN, Mehendale PC, Gadgoli CH (2009). Evaluation of wound healing activity of root of Mimosa pudica. J Ethnopharmacol.

[157] Bum EN, Dawack DL, Schmutz M, Rakotonirina A, Rakotonirina SV, Portet C (2004). Anticonvulsant activity of *Mimosa pudica* decoction. Fitoterapia.

[158] Joseph B, George J, Mohan J (2013). Pharmacology and traditional uses of Mimosa pudica. IJPSDR.

[159] Li YX, Zhu JX, Yang H, Yuan K

[160] Gopalakrishnan L, Doriya K, Kumar DS (2016). *Moringa oleifera*: A review on nutritive importance and its medicinal application. Food Sci Hum Wellness.

[161] Popoola JO, Obembe OO (2013). Local knowledge, use pattern and geographical distribution of *Moringa oleifera* Lam. (Moringaceae) in Nigeria. J Ethnopharmacol.

[162] Oluduro AO (2012). Evaluation of antimicrobial properties and nutritional potentials of *Moringa oleifera* Lam. leaf in South-Western Nigeria. Mal J Microbiol.

[163] Mensah JK, Ikhajiagbe B, Edema N, Emokhor J (2012). Phytochemical, nutritional and antibacterial properties of dried leaf powder of Moringa oleifera (Lam) from Edo North Province, Nigeria. J Nat Prod Plant Resour.

[164] Ogbunugafor H, Eneh F, Ozumba A, Igwo-Ezikpe M, Okpuzor J, Igwilo I (2011). Physico-chemical and antioxidant properties of *Moringa oleifera* seed oil. Pak J Nut.

[165] Ghasi S, Nwobodo E, Ofili JO (2000). Hypocholesterolemic effects of crude extract of leaf of *Moringa oleifera* Lam in high-fat diet fed wistar rats. J Ethnopharmacol.

[166] Pari L, Kumar NA (2002). Hepatoprotective activity of *Moringa oleifera* on antitubercular drug-induced liver damage in rats. J Med Food.

[167] Al-Malki AL, Rabey HAE (2015). The antidiabetic effect of low doses of *Moringa oleifera* Lam. seeds on streptozotocin induced diabetes and diabetic nephropathy in male rats. Biomed Res Int.

[168] Gupta R, Mathur M, Bajaj VK, Katariya P, Yadav S, Kamal R (2012). Evaluation of antidiabetic and antioxidant activity of *Moringa oleifera* in experimental diabetes. J Diabetes.

[169] Abubakar MS, Musa AM, Ahmed A, Hussaini IM (2007). The perception and practice of traditional medicine in the treatment of cancers and inflammations by the Hausa and Fulani tribes of Northern Nigeria. J Ethnopharmacol.

[170] Nwauche TK, Nwosu UG, Ighorodje-Monago C (2017). Hypoglycemic Activity of the aqueous root-back extract of Olax Mannii on diabetic induced albino rats. IJIRAS.

[171] Shehu A, Magaji MG, Yau J, Ahmed A (2017). Ethno-botanical survey of medicinal plants used for the management of depression by Hausa tribes of Kaduna State, Nigeria. J Med Plants Res.

[172] Fankam AG, Kuete V, Voukeng IK, Kuiate JR, Pages J (2011). Antibacterial activities of selected Cameroonian spices and their synergistic effects with antibiotics against multidrug-resistant phenotypes. BMC Complement Altern Med.

[173] Sule M, Hassan H, Pateh U, Ambi A (2011). Triterpenoids from the leaves of* Olax mannii* Oliv. Nig J Basic Appl Sci.

[174] Sule M, Haruna A, Pateh U, Ahmadu A, Ambi A, Sallau M (2005). Phytochemical investigation of leaf, fruit and root bark of Olax manni Oliv. Olacaceae Chem Class J.

[175] Udobi CE, Onaolapo JA (2009). Phytochemical analysis and antibacterial evaluation of the leaf stem bark and root of the African locust bean (Parkia biglobosa). J Med Plants Res.

[176] Tala VRS, Silva VCd, Rodrigues CM, Nkengfack AE, Santos LCd, Vilegas W (2013). Characterization of Proanthocyanidins from Parkia biglobosa (Jacq.) G. Don. (Fabaceae) by Flow Injection Analysis — Electrospray Ionization Ion Trap Tandem Mass Spectrometry and Liquid Chromatography/Electrospray Ionization Mass Spectrometry. Molecules.

[177] Adaramola T, Ariwaodo J, Adeniji K (2012). Distribution, phytochemistry and antioxidant properties of the Genus* Parkia* R.br.(Mimosaceae) in Nigeria. Int J Pharmacognosy Phyt Res.

[178] Komolafe K, Olaleye TM, Omotuyi OI, Boligon AA, Athayde ML, Akindahunsi AA (2014). In vitro antioxidant activity and effect of *Parkia biglobosa* bark extract on mitochondrial redox status. J Acupunct Meridian Stud.

[179] Ayo-Lawal R, Sibuyi N, Ekpo O, Meyer M, Osoniyi O (2021). Investigation of the anti-cancer and apoptotic properties of aqueous extract from fermented African locust bean seeds. Food Res.

[180] Tringali C, Spatafora C, Longo OD (2000). Bioactive constituents of the bark of *Parkia biglobosa*. Fitoterapia.

[181] Dwivedi S, Dwivedi A, Dwivedi S (2008). Folk lore uses of some plants by the tribes of Madhya Pradesh with special reference to their conservation. Ethnobot Leaflets.

[182] Rashmi G, Jaya P, Hardik P, Bhumi M, Shivani A (2010). *Peristrophe bicalyculata* - A Review. Pharm J.

[183] Janakirama N, Johnson M, Sahaya SS (2012). GC–MS analysis of bioactive constituents of Peristrophe bicalyculata (Retz.) Nees. (Acanthaceae). Asia Pac J Trop.

[184] Abdulazeez A, Awasum C, Dogo Y, Abiayi P (2010). Effect of* Peristrophe bicalyculata* on Blood Pressure, Kidney and Liver Functions of Two Kidney One Clip (2K1C) Hypertensive Rats. Br J Pharmacol Toxicol.

[185] Nabèrè O, Samson G, Adama H, Moussa C, Eric SP, Aminata NP (2012). Antioxidant and anticancer activities of polyphenolic compounds from three Acanthaceae medicinal species from Burkina Faso. Int J Phytomed.

[186] Ashokan K, Muthuraman MS (2011). Anticancer studies on Orthosiphon pallidus Royle and Peristrophe bicalyculata Nees. J Pharm Res.

[187] Tanavade SS, Naikwade NS, Chougule D (2012). In vitro anticancer activity of ethanolic extracts of Peristrophe Bicaliculata Nees. International Journal of Chemical Science.

[188] Rani A, Sharma A (2013). The genus Vitex: A review. Pharmacogn Rev.

[189] Agbafor KN, Nwachukwu N (2011). Phytochemical Analysis and Antioxidant Property of Leaf Extracts of Vitex doniana and *Mucuna pruriens*. Biochem Res Int.

[190] Abiodun O, Gbotosho G, Ajaiyeoba E, Happi T, Falade M, Wittlin S (2011). *In vitro* antiplasmodial activity and toxicity assessment of some plants from Nigerian ethnomedicine. Pharm Biol.

